# Comprehensive transcriptomic analysis to identify biological and clinical differences in cholangiocarcinoma

**DOI:** 10.1002/cam4.5719

**Published:** 2023-03-20

**Authors:** Marco Silvestri, Trung Nghia Vu, Federico Nichetti, Monica Niger, Serena Di Cosimo, Filippo De Braud, Giancarlo Pruneri, Yudi Pawitan, Stefano Calza, Vera Cappelletti

**Affiliations:** ^1^ Department of Applied Research and Technological Development Fondazione IRCCS Istituto Nazionale dei Tumori di Milano Milan Italy; ^2^ Unit of Biostatistics, Department of Molecular and Translational Medicine University of Brescia Brescia Italy; ^3^ Department of Medical Epidemiology and Biostatistics Karolinska Institutet Stockholm Sweden; ^4^ Department of Medical Oncology Fondazione IRCCS Istituto Nazionale dei Tumori di Milano Milan Italy; ^5^ Computational Oncology Group, Molecular Precision Oncology Program National Center for Tumor Diseases (NCT) and German Cancer Research Center (DKFZ) Heidelberg Germany; ^6^ Department Pathology and Laboratory Medicine Fondazione IRCCS Istituto Nazionale dei Tumori Milan Italy

**Keywords:** bioinformatics, cholangiocarcinoma, next generation sequencing, transcriptomics, tumor‐infiltrating immune cells

## Abstract

**Background:**

Cholangiocarcinoma (CC) is a rare and aggressive disease with limited therapeutic options and a poor prognosis. All available public records of cohorts reporting transcriptomic data on intrahepatic cholangiocarcinoma (ICC) and extrahepatic cholangiocarcinoma (ECC) were collected with the aim to provide a comprehensive gene expression‐based classification with clinical relevance.

**Methods:**

A total of 543 patients with primary tumor tissues profiled by RNAseq and microarray platforms from seven public datasets were used as a discovery set to identify distinct biological subgroups. Group predictors developed on the discovery sets were applied to a single cohort of 131 patients profiled with RNAseq for validation and assessment of clinical relevance leveraging machine learning techniques.

**Results:**

By unsupervised clustering analysis of gene expression data we identified both in the ICC and ECC discovery datasets four subgroups characterized by a distinct type of immune infiltrate and signaling pathways. We next developed class predictors using short gene list signatures and identified in an independent dataset subgroups of ICC tumors at different prognosis.

**Conclusions:**

The developed class‐predictor allows identification of CC subgroups with specific biological features and clinical behavior at single‐sample level. Such results represent the starting point for a complete molecular characterization of CC, including integration of genomics data to develop in clinical practice.

## 1 INTRODUCTION

Cholangiocarcinoma (CC) is a rare cancer arising from the biliary tree.^1^ Based on the anatomical location, CC is classified as intra‐ (ICC) and extrahepatic (ECC), the latter comprising perihilar (pCC) and distal (dCC),^2^ with biological differences and implications for clinical management.^3^ Regardless of its location, CC is an aggressive malignancy, with limited therapeutic options and a dismal prognosis, 5‐year overall survival being 5%–10%.^4^ Due to steady increase of its incidence, from 0.3 to 6 (depending on geographical area): 100,000 yearly over the last decade, CC—once considered a rare cancer—is now becoming a global health problem. Surgery represents the only curative treatment, but the frequent absence of symptoms in early stages results in most cases in late diagnosis, when the disease is inoperable. In the advanced setting, the combination of cisplatin and gemcitabine has long represented the standard of care producing a median PFS of 8 months.^5^ A new option is adding immunotherapy, that is, durvalumab, which further reduces the risk of progression by 25%, and increases survival by 20%. Yet more than half of patients with inoperable CC continue to die within a year of diagnosis.^6^

In recent years, the widespread use of next‐generation sequencing technologies revealed a complex genomic, epigenomic, and transcriptomic landscape of CC, leading to the identification of distinct molecular subtypes and new targets for molecularly informed treatments. Among these, clinical trials demonstrated significant activity for drugs targeting *IDH1*,^7^ *FGFR2*,^8^, ^9^, ^10^ *BRAF*,^11^ and *HER2*,^12^ which are now entering clinical practice. Nonetheless, the majority of CC does not harbor alterations in these genes,^5^, ^13^ making the identification of new molecular biomarkers an urgent need.

In this light, a series of gene expression studies using transcriptomic technologies on primary CC tumors attempted to elucidate the mechanistic insights of CC and to identify transcriptomic subtypes with a predictive and prognostic relevance. Considering 149 ICC cases, Sia and colleagues demonstrated the presence of two classes, named as proliferation‐ and inflammation‐related, associated with up‐regulation of EGF, RAS, AKT, MET signaling, and immune response‐related pathways, respectively.^14^ In another study, Andersen and colleagues identified two prognostic subtypes and demonstrated the therapeutic potential of tyrosine kinase inhibition in CC cell lines with activated EGFR and HER2 signaling pathways.^15^ Regarding ECC tumors, Montal and colleagues identified in 189 patients the presence of four classes characterized by different transcriptomic and genomic patterns related to metabolic, proliferation, mesenchymal, and immunological processes with comparable prognosis in terms of overall survival.^16^ Finally, Nakamura and colleagues, with a comprehensive analysis of ICC, ECC and including also gallbladder tumors, demonstrated the presence of four subgroups defined by specific gene expression and correlated genomic profile, associated with clinical outcome.^17^

Despite these results represented a significant improvement in the biological understanding of CC, their clinical implications are still limited. This is due not only to the limited application of RNA‐sequencing in clinical practice, but also to the fact that all these classifications were generated from different and small cohorts and have not been compared and unified, thus limiting their applicability. Indeed, a comprehensive transcriptomic analysis of ICC and ECC for obtaining biological insights and prognostic information across different studies is still lacking. Availability of a classification combining several single studies with small patient cohorts is likely to deliver more solid data on ICC and ECC transcriptomic subtypes possibly identifying new druggable pathways.

In the present work, we aim at filling the gap by providing a comprehensive transcriptomic analysis of ICC and ECC tumor tissues derived from all the public cohorts available to date, with the final goal of generating a definitive and uniformed gene expression‐based classification of CC and of identifying lists of markers with prognostic relevance.

## 2 MATERIALS AND METHODS

### 2.1 Retrieval of public ICC and ECC gene expression datasets and definition of discovery and validation sets

Gene expression profiles were collected from eight public datasets, including 674 patients, that is, 442 (65.6%) ICC and 232 (34.4%) ECC with no previous history of hepatitis or fluke infection, and generated by microarray (6) or RNA sequencing (RNAseq, 2). Overall, 126 of 664 (19%) were from fresh frozen (FF) tumor tissues, while 538 (81%) were from formalin‐fixed paraffin embedded (FFPE) specimens. For all the downstream analyses, seven datasets were used for discovery; whereas the EGAD00001001693 dataset was used for validation proven that it was the unique with complete information on patient survival (Table 1).

**TABLE 1:** Collection of gene expression dataset considered for ICC and ECC discovery and validation sets.

Histotype	Dataset ID	Specimen	Reference	No. of samples	Platform	Discovery (D)
						Validation (V)
ECC	GSE132305	FFPE	Montal et al.^16^	182	Microarray (Affymetrix)	D
ICC	GSE32225	FFPE	Sia et al.^14^	141	Microarray (Illumina)	D
ICC	GSE26566	FF	Andersen et al.^15^	103	Microarray (Illumina)	D
ICC, ECC	GSE89749	FFPE	Jusakul et al.^18^	58	Microarray (Illumina)	D
ICC, ECC	TCGA‐CHOL	FFPE	Farshidfar F. et al., ^19^Cell Reports 2017	36	RNAseq	D
ICC	GSE32879	FF	Oishi N. et al., ^20^ Hepatology 2021	16	Microarray (Affymetrix)	D
ICC	GSE57555	FF	Murakami Y. et al., ^21^ Scientific Reports 2015	7	Microarray (Agilent)	D
ICC, ECC	EGAD00001001693	FFPE	Nakamura et al.^17^	131	RNAseq	V

Abbreviations: ECC, extrahepatic cholangiocarcinoma; FF, fresh frozen; FFPE, formalin‐fixed paraffin embedded; ICC, intrahepatic cholangiocarcinoma.

### 2.2 Data processing

For the training set, transcript quantification data of the TCGA‐CHOL cohort (level 3) obtained using RSEM (level 3) were downloaded from Firebrowse database (accession date: January 2022). TPM values followed by quantile normalization and log2 transformation were considered. All the microarray data were retrieved from GEO using a bespoke R pipeline. Considering Illumina microarray dataset, robust‐spline normalization followed by log2 scaling were used to normalize gene expression values. Affymetrix and Agilent datasets did not require any additional normalization after downloaded from GEO database.

**FIGURE 1:**
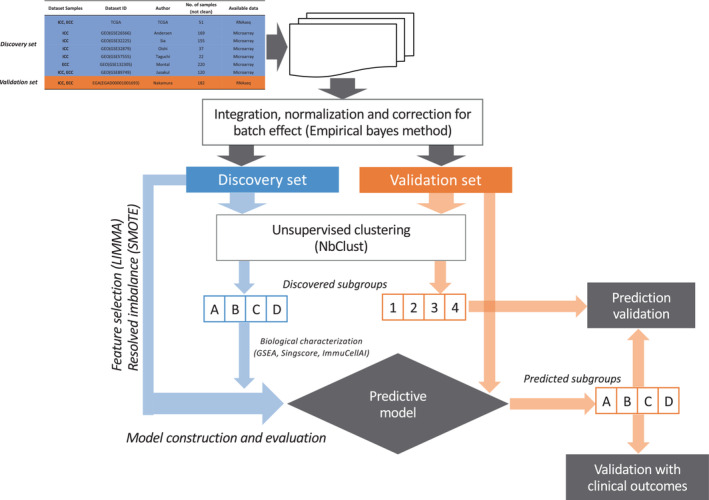
Workflow of the analysis. The pipeline reports all the steps used in the present works, from data collection to the clinical evaluation of the defined intrahepatic cholangiocarcinoma and extrahepatic cholangiocarcinoma subgroups in validation set.

Regarding the validation set, RNAseq data were downloaded from the EGA repository after the accession permission released by ICGC consortium (application number: DACO‐6992). Raw sequencing data (fastq format) were trimmed to remove low‐quality bases and adapters using trimmomatic (version 0.39),^22^ quality checked using FastQC (http://www.bioinformatics.babraham.ac.uk/projects/fastqc) and aligned to human reference genome (hg19) using STAR (version 2.7.9a).^23^ After alignment quality control by bedtools (version 2.25.0)^24^ and qualimap (version 2.2.2‐dev),^25^ counting of reads aligned over exonic features for gene expression quantification was performed by RSEM (1.3.1).^26^ TPM values related to each gene were considered and submitted to quantile normalization and log2 scaling. All the processing (discovery set) and post‐processing analysis (validation set) were performed with R software (https://www.R‐project.org/, version 4.1.1, see section “list of R packages”). Figure 1 reports in detail all the steps of the pipeline adopted in the present work.

### 2.3 Data integration and unsupervised clustering analysis of ICC and ECC discovery sets

Normalized gene expression data of each dataset included in the ICC and ECC discovery sets were merged and only common transcripts (*n* = 13,228) were considered. Then, quantile normalization followed by batch adjustment based on empirical Bayes method^27^ were performed to make data comparable and remove batch effect associated with the different samples source (FF, FFPE) and platforms to obtain gene expression profiles. GTEx database (accession date: February 2022) was interrogated to remove 386 liver‐specific transcripts associated to normal tissues. A filtering step was performed using a custom made pipeline to reduce the number of features in the ICC (*n* = 1358) and ECC (*n* = 676) cohorts, respectively. In particular, the method allows to identify genes that drive biological heterogeneity in a dataset decomposing the total variance of each gene into its biological and technical components by fitting a trend to the endogenous variances.^28^ For each gene, the fitted value of the trend is an estimate of the technical component while the biological component is retrieved by subtracting the fitted value from the total variance. For the ICC training set, genes with significant biological component were selected using a criteria based on a FDR <0.05. Due to the small number of datasets in the ECC training set, the filtering step was performed separately for each dataset (block) and only genes with FDR ≤0.1 in each block were selected.

Detection of distinct subgroups within the ICC and ECC training set was performed by unsupervised clustering analysis through NbClust tool.^29^ In particular, 19 out 30 indices were considered to validate the number of the clusters (*n* min = 2, *n* max = 6) along with euclidean distance and Ward's linkage method.^30^

### 2.4 Differential expression analysis, functional enrichment analysis, and evaluation of immune cells component

Within ICC and ECC discovery sets, differential expression analysis between subgroups were performed using gene level linear models with moderated *t*‐test (LIMMA).^31^ *t*‐statistic values were used to rank gene list for the gene set enrichment analysis by GSEA^32^ considering HALLMARK and C_2_ canonical pathway database.^33^ For each cluster comparison, significant up‐regulated/down‐regulated pathways were selected according to thresholds of *p*‐value <0.05 and *p*‐value <0.01 for HALLMARK and C_2_ canonical pathway gene sets, respectively. Singscore tool,^34^ a single sample scoring method, was used to evaluate the enrichment of specific CC pathways described by Banales and colleagues^35^ within each ICC and ECC subgroups. *t*‐test statistic was applied to detect significant differences in terms of enrichment score. ImmuCellAI^36^ was used to evaluate immune cell component within each ICC and ECC subgroups based on the deconvolution of gene expression profiles of 24 immune cell. *t*‐test statistics was applied to detect significant difference between subgroups in terms of immunological infiltration score.

### 2.5 Building of ICC and ECC predictors

Starting from differential expression analysis by LIMMA, two different approaches were adopted to define the genes to be considered for the predictor establishment in ICC and ECC discovery set. For ICC cohort, genes with a log2 fold change (log2FC) <−1 and >1 and FDR <0.1 values within each comparison were selected (*n* = 19). Considering the ECC cohort, among the top 60 differentially expressed genes for each group based on *t*‐statistic value, only the transcripts with FDR <0.25 in all subgroups were selected (*n* = 21). The SMOTE (Synthetic minority over‐sampling technique) algorithm,^37^ was applied to account for sample imbalance between the subgroups within ICC and ECC cohort. Three different machine learning algorithms were considered for the establishment of the predictors: k‐nearest neighbors (KNN),^38^ support vector machine (SVM),^39^ and fast unified random forest (rFSRC).^40^ In order to evaluate the KNN and SVM methods, training and testing‐sets were retrieved by subsampling the 60% and 40% of ICC and ECC discovery sets, respectively. Moreover, a 10‐fold cross validation was applied. For rFSCR algorithm, bootstrap without replacement was considered as resampling method. ROC curves and AUC were used to visualize and evaluate the performance of the classifiers for each ICC and ECC subgroup.

### 2.6 Prediction and evaluation of biological subgroups in ICC and ECC validation sets

Starting from the same genes considered for the unsupervised clustering analysis in discovery set (*n* = 13,228), NbClust algorithm was applied to validation sets. In particular, 19 out of 30 indices were considered to validate the number of the clusters along with euclidean distance and Ward's linkage method.^30^ For ICC validation set the minimum and maximum number of groups admitted were two and six while for ECC, due to the low number of samples (*n* = 29), the range was set to two and five. At the same time, predictors based on rFSRC algorithm were applied to predict the presence of the four biological subgroups in ICC and ECC validation sets. Diagonal dominant matrix^41^ approach along was used to understand the concordance between NbClust‐based and predictors‐based classification. Moreover, unsupervised clustering analysis based on the median expression of the 19 and 21 genes of each biological sublclasses identified by the two classification methods were applied in ICC and ECC validation sets. Euclidean distance and Ward.D linkage were considered for the analysis.

### 2.7 Survival analysis

Survival analysis methods were used to analyze overall survival (OS) and relapse free survival (RFS). OS was calculated from the date of disease diagnosis to death or last follow‐up, while RFS was calculated from the date of disease diagnosis to the first event (i.e., disease relapse or death). Patients were divided into biological subgroups returned by rFSRC predictor and unadjusted *p*‐values were calculated using log‐rank test considering <0.05 as threshold of statistically significance. Considering ICC cohort, three patients were excluded from the survival analysis due to missing OS and PFS info.

### 2.8 List of R packages

1. WGCNA

2. UBL

3. tidyr

4. sva

5. singscore

6. RColorBrewer

7. randomForestSRC

8. plyr

9. org.Hs.eg.db

10. NMF

11. NbClust

12. multiROC

13. lumi

14. limma

15. lattice

16. irr

17. GSA

18. gplots

19. ggthemes

20. ggpubr

21. ggplot2

22. ggfortify

23. GEOquery

24. FunCluster

25. fgsea

26. factoextra

27. edgeR

28. dplyr

29. DBI

30. data.table

31. ComplexHeatmap

32. circlize

33. caret

34. Biobase

## 3 RESULTS

### 3.1 Integration of gene expression data from different platforms for the establishment of discovery set

A total of 340 ICC and 203 ECC patients profiled for gene expression data with no previous history of hepatitis or fluke infection were collected from seven different datasets and considered as discovery dataset. Overall, the intersection of the different platforms used to characterize each sample allowed to obtain the expression profile of 13,228 genes. The application of preliminary steps for sample normalization and batch effect adjustment lead to a higher correlation between each sample in the cohorts (range: 0.5–1 for ICC; range: 0.6–1 for ECC) and to efficient batch correction (Figures S1 and S2).

Liver‐specific genes (*N* = 386) obtained from GTEx database were removed to avoid contamination given by transcripts non biologically associated to CC.^16^ Moreover, the additional filtering step performed based on the mean expression and variance of each genes allowed to select 1358 and 676 genes in ICC and ECC cohort, respectively (Figure S3).

### 3.2 Unsupervised clustering analysis identifies distinct biological subgroups in ICC and ECC cohorts

**FIGURE 2:**
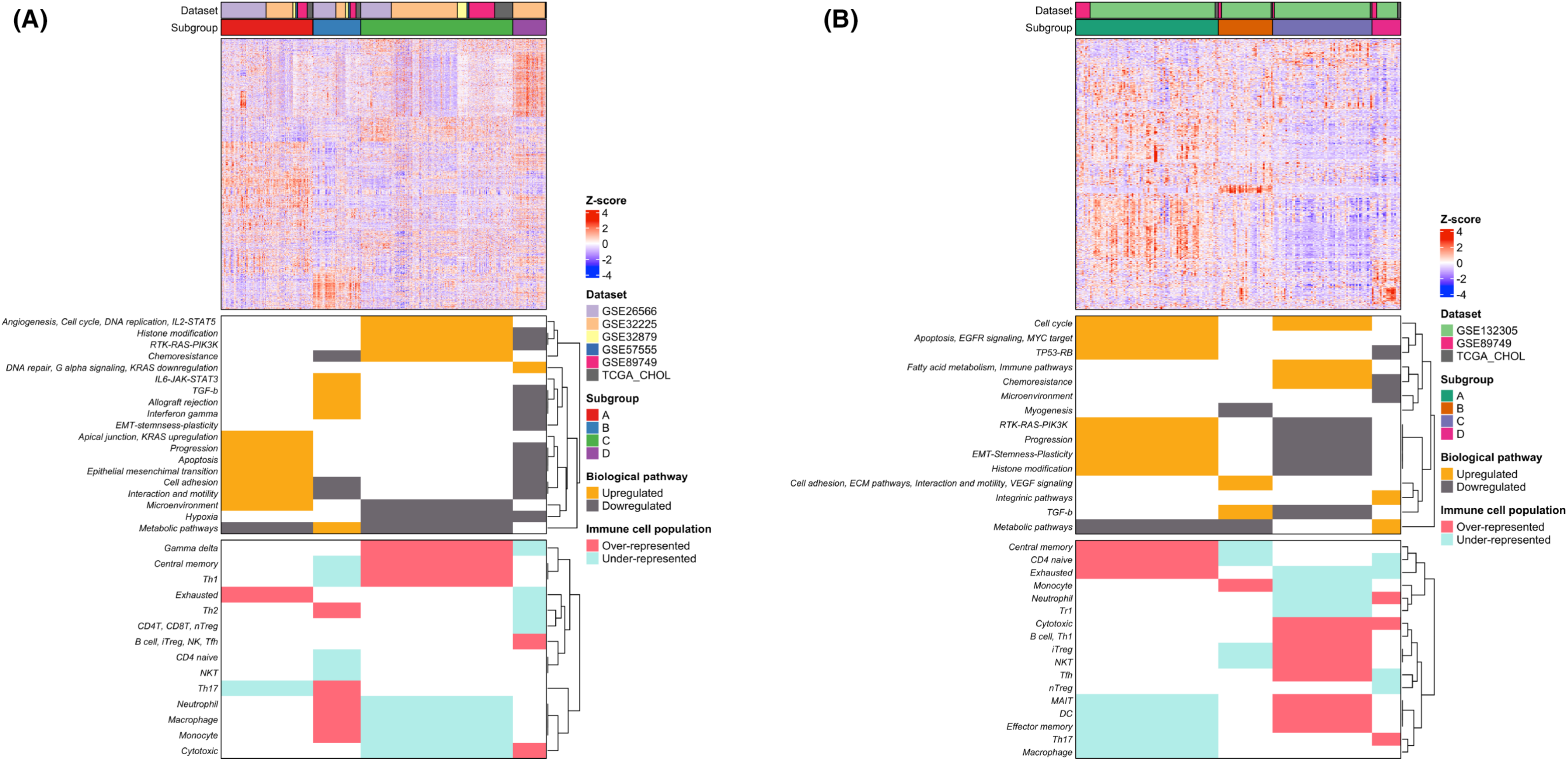
Identification of intrahepatic cholangiocarcinoma (ICC) and extrahepatic cholangiocarcinoma (ECC) distinct biological subgroups. Unsupervised clustering analysis using NbClust identified four subgroups in both ICC (A) and ECC (B) discovery set. The heatmaps reports samples on the column and genes on the rows. For each sample, dataset of origin and the cluster group membership are reported as color bars on the top of the heatmap. In order to evaluate the biological characteristics associated to each subgroup, GSEA, Singscore, and ImmuCellAI were applied on ICC and ECC cohort. Upregulated/downregulated pathways and Over‐Under represented immune cell populations (rows) associated to each ICC and ECC subclass (columns) were reported as annotation below the heatmaps.

Unsupervised clustering analysis using NbClust led to the identification of four subgroups within both the ICC (*N* = 340, Table S1) and ECC (*N* = 203, Table S2) cohort (Figure 2A,B), with no evidence of batch effect related to the different datasets forming each cohort.

To investigate the presence of distinct biological and immunological features of the newly defined subgroups, we collected the differentially expressed genes subgroups of each group. Results highlighted significant differences between each subgroup, with the distribution of up‐ and down‐regulated genes that varied among the ICC and ECC cohort (Figure S4). To further understand the unique biological traits of the subgroups, enrichment analysis along with the evaluation of immunological components were performed.

Regarding the ICC cohort, up‐regulated pathways/signatures characterized distinct biological features of each subgroup, mainly related to apoptosis and progression (subgroup A), metabolism and TGF‐ß (subgroup B), cell cycle and DNA replication (subgroup C), DNA repair and KRAS down‐regulation (subgroup D). Interestingly, the evaluation of the immunological components revealed the presence of immune cell infiltration in all samples, with subgroup A being enriched only with exhausted T cells (Figure 2A; Figures S5–S8). The enrichment analysis on samples of the ECC cohort also showed distinct biological traits associated to each subgroup, related to EGFR and MYC target (subgroup A), ECM and cell adhesion/interaction/motility (subgroup B), immune pathways and cell‐cycle (subgroup C), and metabolic pathways (subgroup D). Moreover, all the subgroups presented different immunological components, with subgroup C being characterized by the highest rate of immune cells infiltration (see Figure 2B and Figures S9–S12).

### 3.3 Supervised classifier to identify the proposed subgroups using gene expression

**FIGURE 3:**
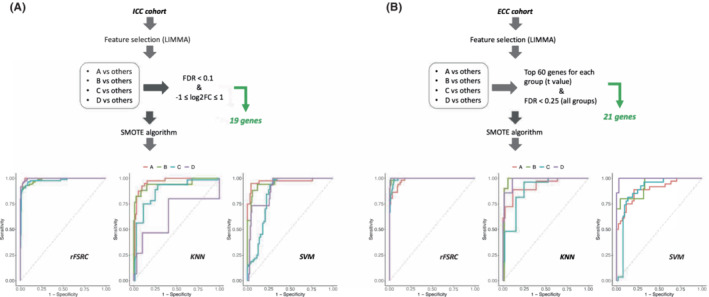
Building of predictors related to the distinct biological subgroups in intrahepatic cholangiocarcinoma (ICC) and extrahepatic cholangiocarcinoma (ECC) cohort. For ICC, class comparison results obtained from LIMMA allowed to select 19 genes specifically associated to each subgroup (FDR <0.1 & −1 ≤ log2FC ≤1) (A). Within ECC, class comparison results obtained from LIMMA allowed to select the top 60 differentially expressed genes based on *t* value. Then, the filtering based on FDR value (FDR <0.25 in all groups) allowed to identify 21 genes specifically associated to each subgroup (B). For both ICC and ECC cohort, synthetic minority over‐sampling technique algorithm was applied to balance the distribution of the samples within subgroups and three different machine learning methods were evaluated for the establishment of the predictor. The ROC curves report specificity and sensitivity values obtained from fast unified random forest, k‐nearest neighbors, and support vector machine algorithms.

The presence of distinct ICC and ECC biological subgroups within the discovery set opened the possibility to build and compare specific predictors to validate our findings in an independent dataset (validation set). For such a purpose, feature selections were performed specifically for ICC and ECC cohorts starting from the differential expression analysis between each subclass. A total of 19 and 21 genes were selected for ICC and ECC samples, respectively and used to build the predictor from training set. Due to imbalance between subgroups, we adopted the SMOTE algorithm in order to oversample the minority class prior to classifier definition on the discovery set. Among the three machine learning algorithms tested, rFSRC showed the best performance with an overall misclassification rate of 0.08 and 0.07 for ICC (Table S3) and ECC (Table S4), respectively. Moreover, rFSRC predictor showed an AUC value of 0.98 (KNN = 0.84; SVM = 0.9) and of 0.99 (KNN = 0.95; SVM = 0.93) for ICC and ECC cohort, respectively. In particular, the classification error associated to each subclass were similar except for group A in the ECC cohort, where a value of 0.17 was obtained (Figure 3).

### 3.4 Identification of ICC and ECC biological subgroups in validation set

To validate and establish the clinical significance of our predictors, an independent dataset composed of 131 patients (102 ICC, 29 ECC) profiled by RNAseq was considered as ICC and ECC validation sets (Table 2). Similarly to the discovery set, all of the patients did not present a previous history of hepatitis or fluke infection. After transcripts quantification, normalization and gene filtering steps, we used a Random Forest‐based classifier to predict the presence of the four specific biological subgroups in both ICC and ECC of the validation set. In the ICC cohort, the subgroups A (*n* = 43) and D (*n* = 1) were characterized by the highest and lowest number of patients, respectively, consistently with the discovery set composition. For the ECC cohort, most of the patients were identified in subgroups A (*n* = 24) and only a few samples were assigned to the B (*n* = 2), C (*n* = 2), and D (*n* = 1) subgroups.

**TABLE 2:** Clinical characteristics of patients in validation set.

Characteristic	ICC *N* (%)	ECC *N* (%)
*Age*		
<67 years	47 (46.1%)	9 (69%)
≥67 years	52 (51%)	20 (31%)
Missing	3 (2.9%)	—
*Clinical tumor size*		
cT1	4 (39.2%)	7 (24.1%)
cT2	47 (46.1%)	10 (34.5%)
cT3	17 (16.6%)	10 (34.5%)
cT4	31 (30.4%)	2 (6.9%)
Missing	3 (2.9%)	—
*Clinical nodal status*		
cN0	67 (65.7%)	22 (75.9%)
cN1	32 (31.4%)	7 0 (24.1%)
Missing	3 (2.9%)	—
*Stage*		
1	4 (39.2%)	10 (34.5%)
2	34 (33.3%)	15 (51.7%)
3	12 (11.8%)	2 (6.9%)
4	49 (48%)	2 (6.9%)
Missing	3 (2.9%)	—
*Relapse*		
Yes	61 (59.8%)	10 (34.5%)
No	38 (37.3%)	19 (65.5%)
Missing	3 (2.9%)	—

Abbreviations: ECC, extrahepatic cholangiocarcinoma; ICC, intrahepatic cholangiocarcinoma.

**FIGURE 4:**
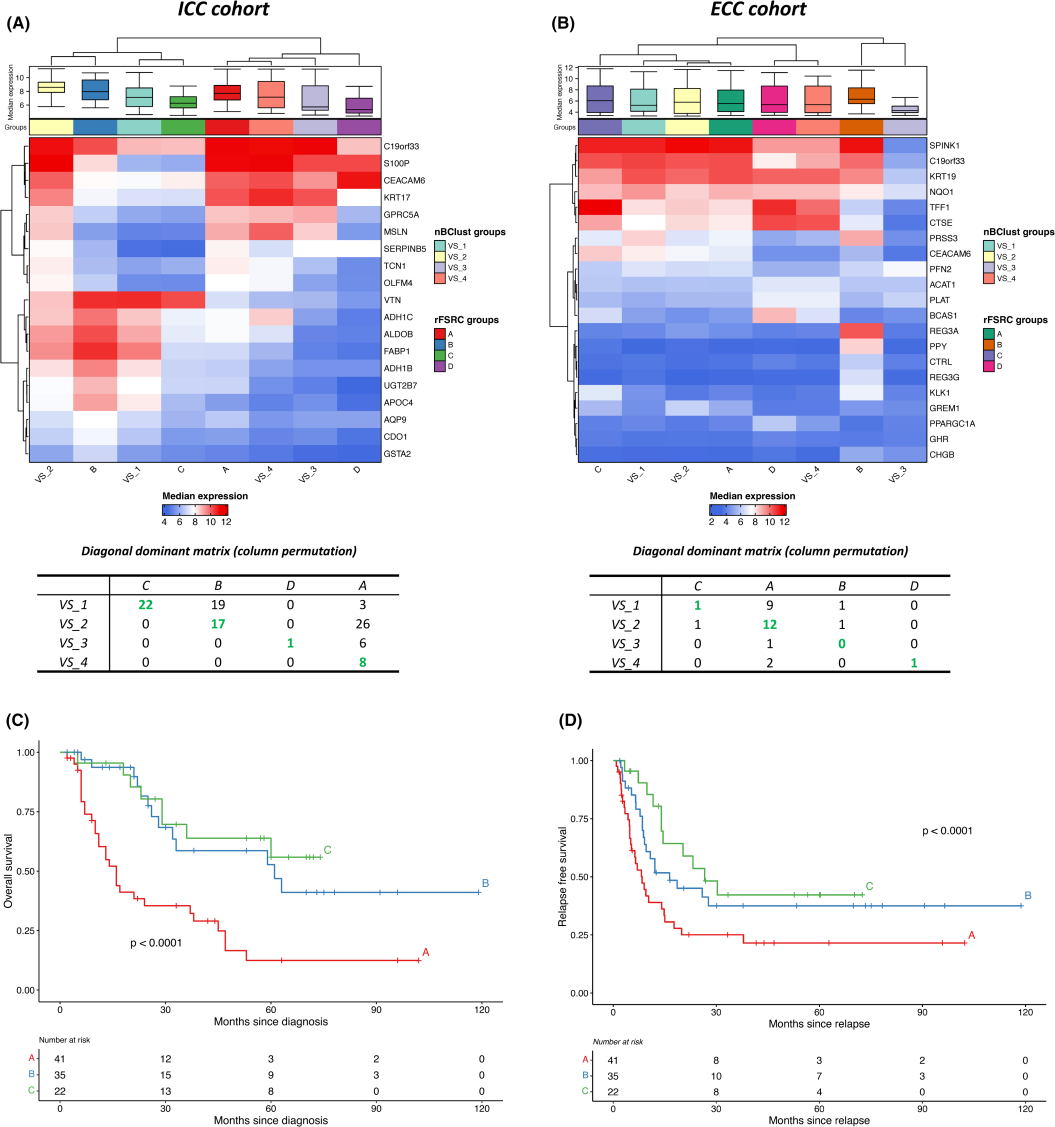
Comparison of predictor‐based and NbClust‐based classification in intrahepatic cholangiocarcinoma (ICC) and extrahepatic cholangiocarcinoma (ECC) validation set and evaluation of the clinical outcome. To evaluate the validity of classification returned by the ICC and ECC predictors, a comparison with subgroups returned by NbClust was performed using diagonal dominant matrix approach and comparing the median expression values associated to each group. For both ICC (A) and ECC (B) cohort, heatmap shows subgroups on the columns and genes on the rows. The subgroup membership and the associated gene expression levels are reported at the top of the heatmap as color bar and box plot, respectively. Diagonal dominant matrix results are shown at the bottom of the heatmap. Overall survival (C) and relapse‐free survival (D) analysis in ICC cohort are represented using the Kaplan‐Meier method. Due to the low number of patients, ECC cohort was not considered for survival analysis.

To validate the subgroups predicted by the supervised classifier, we compared them to the subgroups discovered by unsupervised clustering analysis on the validation sets, which were independent from the discovery sets. The method was applied on the same genes (*N* = 1358 for ICC; *N* = 676 for ECC) used to detect the subgroups in the discovery dataset. In order to match the group labels provided by the unsupervised clustering to the predicted subgroup classes, we tabulated the unsupervised labels versus the predictor‐based groups is a 4 × 4 matrix and searched for the dominant diagonal matrix (the matrix that maximize the numbers along the main diagonal) using column permutation and we evaluated the median expression of genes within each specific subgroup. Considering the ICC cohort, the column permutation showed specific concordance between the subgroups identified by the two methods, as strongly demonstrated by the association between VS_1—C and VS_2—B classes. Interestingly, the 19 genes considered for the classification presented peculiar patterns of up‐regulation (e.g., VTN, ADH1C, ALDOB, FABP1 in VS_2—B) and down‐regulation (e.g., TCN1, OLFM4, VTN, ADH1C in VS_3—D) demonstrating the presence of biological differences between the subgroups (Figure 4A). Within ECC cohort, the column permutation highlighted specific association between the subgroups from predictor‐based and NbClust‐based approaches except for groups B and VS_3, where the low number of patients in validation set affects the degree of concordance. Similar to ICC, the comparison of median expression showed patterns of up‐regulation and down‐regulation for the 21 genes, also for the B‐VS_3 groups (e.g., GREM1, PPARGC1A, GHR, and CHGB) (Figure 4B).

### 3.5 Identified ICC biological subgroups are associated with clinical outcome

In order to understand the clinical relevance of the expression subgroups, OS and RFS were evaluated in the ICC validation cohort. While considering OS, subgroup A showed a significantly worse prognosis compared to subgroups B and C (*p*‐value <0.0001, Figure 4C). Similarly, the analysis of RFS demonstrated that subgroup A was characterized by patients with the shorter RFS (Figure 4D). These results support a link between biological characteristics of the primary tumors and the prognosis. Indeed, patients within subgroup A had the worst prognosis and RFS with tumors characterized by KRAS up‐regulation, epithelial‐to mesenchymal‐transition, and apoptosis and by the absence of immune cell infiltration. On the contrary, despite the presence of several cancer associated up‐regulated pathways such as RTK‐RAS‐PIK3K and TGF‐ß, tumors belonging to subgroups B and C were characterized by a strong immunological component, improving patients' prognosis both in terms of OS and RFS (Figure 4C,D). Unfortunately, the low number of patients prevented a similar survival analysis for ECC subgroups.

## 4 DISCUSSION

In the present study, we report the identification of four biologically‐distinct and prognostically‐relevant ICC and of ECC subgroups characterized by a unique gene expression profile and generated a parsimonious class predictor for their identification.

The identified subgroups are characterized by the enrichment of specific molecular pathways such as EMT, IL6, RTK‐RAS‐PIK3K, and DNA repair in ICC tumors and EGFR, VEGF signaling immune, and integrine‐pathways in ECC. Immune cell infiltrates are peculiar and distinct among subgroups supporting their unique biological profiles and possible treatment implications. Finally, the analysis of the overall and relapse‐free survival highlights a significant association between biological subgroups and clinical outcome in the ICC cohort, identifying one specific subclass characterized by poor prognosis.

Several studies aimed at characterizing cholangiocarcinoma using integrative molecular analyses.^14^, ^16^, ^17^, ^18^ However, a comprehensive transcriptomic analysis of ICC and ECC derived from different patient cohorts for obtaining biological insights and prognostic information was still lacking. Herein, at variance with previous studies, we have collected and combined for the first time all public gene expression datasets available at study cut‐off (March 2022) in a unique cohort split into discovery and validation sets.

When collecting large numbers of samples derived from different studies the careful integration of the gene expression datasets derived from multiple high throughput platforms is mandatory. The choice of the optimal method for integration of expression data represents a critical step impacting on the final relevance of findings. Herein, we did not only face the need of integrating heterogeneous sample sources (FF, FFPE), but also gene expression data derived from different platforms ranging from microarrays to RNAseq. The use of cross‐platform analysis was a crucial step that allowed improving the classification and identification of tumor phenotypes not only when considering different type of microarrays^42^ but also when integrating them with RNAseq technology.^43^, ^44^, ^45^

The building of ICC and ECC discovery sets derived by distinct datasets was made possible by resorting to empirical Bayesan methodology (ComBat), to remove batch effects associated with each dataset, a mandatory step as already reported.^46^

In a data‐driven approach, such as ours, the choice and careful evaluation of clustering methods is critical. In the current study, the application of NbClust was instrumental for the robust definition of the optimal number of clusters within ICC and ECC of the discovery sets.

In fact, the robustness of the identified subgroups was confirmed by statistical considerations, but was also fully supported by the biologically distinct traits of each identified cluster. For digging deeply into the biology of clusters, the gene set enrichment and single sample scoring analyses were complemented by the evaluation of immune cell infiltration. The biological singularity of each cluster identified by unsupervised classification was indeed further supported by the different profiles of immune infiltrate associated with each subgroup.

In detail, beside to the presence of distinct canonical CC gene signaling as TGF‐b, RTK‐RAS‐PIK3K,^35^ cell cycle, and the up‐regulation/down‐regulation of biological pathways related to cell cycle and metabolic processes, our results showed different immune cell populations within subgroups identifying in both ICC and ECC a subgroup (subgroup A) of tumors characterized by an immune exhausted tumor microenvironment. In ICC, subgroup A displayed a gene expression profile suggesting epithelial‐mesenchymal transition with modifications of cell–cell interaction, adhesion and motility accompanied by KRAS up‐regulation, whereas in ECC immune exhaustion was associated with proliferation and EGFR signaling. For ECC tumors it is interesting to note the strong immune infiltration detected in subgroup C, which was characterized by the presence of nine different immune cell populations. Indeed, in this latter group GO identified up‐regulation of immune pathways, but also pathways linked to chemoresistance.

Having identified interesting subgroups of ICC and ECC tumors endeavored with possible treatment implications, we set to find a way to apply those results to the real‐world daily practice.

The first step was the building and the validation of a classifier with a reduced number of genes. To such purpose, short gene signatures (ICC *N* = 19; ECC *N* = 21) were extracted from differential expression analysis between subgroups taking advantage from the SMOTE algorithm for adjusting sample number unbalances. These steps were necessary to obtain a more accurate establishment of the ICC and ECC predictors after the test of three different machine learning algorithms. The rFSRC method showed the best performance in cohorts, given the low error rate in classification (misclassification error = 0.08).

As a first step in the validation process, we compared the predictor‐based and the NbClust‐based classifications by using diagonal dominant matrix and evaluating the median gene expression levels demonstrating the presence of the four biological subgroups in both cohorts. Interestingly, the two methods correlate well despite the hugely different number of genes considered (Predictor: ICC = 19, ECC = 21; NbClust: ICC = 13,328, ECC = 676).

The use of gene expression classification questions the use of the same type of therapeutic approach for all CC since both among ECC and ICC clear differences on activation of pathways have been observed. Indeed, survival analyses performed in the ICC validation set disclosed an association between predicted subclass and the patient's clinical outcome, with patients whose tumors fell in subgroup A showing the worst prognosis in terms of overall and relapse‐free survival. Due to the low number of samples, it was not possible to perform the analysis with a statistical significance in the ECC cohort.

ICC patients with tumors classified as subgroup A represent 43% and their poor prognosis is supported by up‐regulation of pathways linked to intracellular signaling and epithelial mesenchymal transition and by an enrichment of exhausted T‐cells in their infiltrate. These patients might therefore respond to checkpoint inhibitors provided that appropriate biomarkers are used since their poor prognosis is strongly determined by such a peculiar type of immune infiltrate.

From the clinical point of view, these data may have relevance and applications in the near future. The recent results from the TOPAZ‐1 trial showed that the addition of the immune checkpoint inhibitor (ICI) durvalumab to standard first‐line chemotherapy significantly improved OS in advanced CC patients.^6^ Given the newness of these results and the small magnitude of the reported benefit, no biomarkers have been validated to identify ICI‐sensitive and ‐resistant tumors. However, molecular studies have highlighted CC‐subgroups with up‐regulation of immune‐related biomarkers indicative of an inflamed tumor‐immune microenvironment, thus potentially sensitive to ICIs.^47^ Similarly, RNAseq methods have been shown able to predict sensitivity both to ICIs and targeted treatments.^48^ Therefore, given the prognostic relevance of our newly defined subgroups and their easy reproducibility, testing their predictive role in clinical trials and real‐world cohorts represents the natural next step.

The integration of different datasets and the building of robust classifiers represent main strengths of our study broadening both biological and clinical implications. Indeed, given the rarity of this tumor entity, here we managed to assemble the largest cohort of CC cases profiled with gene expression sequencing, providing a unified and reproducible tool to define CC subgroups. This effort lays the groundwork for future application in prospective specific cohorts, providing a unified schema for CC patient stratification.

Our work is still preliminary and has therefore limitations. A major limitation involves the low number of patients with ECC tumors. This did not hinder identification and validation of ECC subgroups, but definitely interfered with evaluation of their prognostic relevance. Nonetheless, the interesting results obtained in ICC imply that our approach levering public datasets is valuable and can be further implemented in the future when more ECC datasets are available. Availability of larger numbers of ECC profiles will also allow searching for differences between pCC and dCC.

So far precision medicine approaches in CC patients mostly relied on genomic alterations ranging from gene fusions to activating or inactivating mutations. Our results open a different perspective suggesting that gene expression data may refine genomic classes offering the opportunity of better tuning target treatments.

Indeed, the transcriptomic analysis of publicly available CC datasets allowed identifying and validating the presence of biologically distinct subgroups associated to clinical outcome and providing clinical insight into the disease. Such results represent the starting point for a complete molecular characterization of CC, with the integration of genomics data that will allow a comprehensive description of the tumor. We foresee that genomic data integration into the transcriptome classification of patients with CC will aid prognostication and support personalized treatment.

## AUTHOR CONTRIBUTIONS

**Marco Silvestri:** Conceptualization (equal); data curation (equal); formal analysis (equal); investigation (equal); methodology (equal); resources (equal); software (equal); validation (equal); visualization (equal); writing – original draft (equal); writing – review and editing (equal). **Trung Nghia Vu:** Data curation (equal); formal analysis (equal); methodology (equal); resources (equal); supervision (equal); writing – review and editing (equal). **Federico Nichetti:** Supervision (equal); visualization (equal); writing – review and editing (equal). **Monica Niger:** Supervision (equal); validation (equal); writing – review and editing (equal). **Serena Di Cosimo:** Supervision (equal); validation (equal); writing – review and editing (equal). **Filippo de Braud:** Supervision (equal); writing – review and editing (equal). **Giancarlo Pruneri:** Supervision (equal); writing – review and editing (equal). **Yudi Pawitan:** Resources (equal); supervision (equal); validation (equal); writing – review and editing (equal). **Stefano Calza:** Conceptualization (equal); data curation (equal); methodology (equal); resources (equal); supervision (equal); visualization (equal); writing – original draft (equal); writing – review and editing (equal). **Vera Cappelletti:** Conceptualization (equal); investigation (equal); methodology (equal); resources (equal); supervision (equal); validation (equal); visualization (equal); writing – original draft (equal); writing – review and editing (equal).

## FUNDING INFORMATION

The research leading to these results has received funding from AIRC under IG2018‐ID.21694 project‐P.I. Cappelletti Vera. M.S. is the recipient of an AIRC fellowship (N. 25,430). The funders had no role in the design of the study; in the collection, analyses, or interpretation of data; in the writing of the manuscript, or in the decision to publish the results.

## CONFLICT OF INTEREST STATEMENT

M.N. is a recipient of travel expenses from Celgene, speaker honorarium from Accademia della Medicina, and Incyte; honoraria from Sandoz, Medpoint SRL and Servier for editorial collaboration. Consultant honoraria from EMD Serono, Basilea Pharmaceutica, Servier, Incyte, and MSD Italia. F.D.B. is a consultant advisory board for Roche, EMD Serono, NMS Nerviano Medical Science, Sanofi, MSD, Novartis, Incyte, BMS, Menarini, Astra Zeneca, Pierre Fabre. F.D.B is a speaker for BMS, Healthcare Research & Pharmacoepidemiology, Merck Group, MSD, Pfizer, Servier, Sanofi, Roche, AMGEN, Incyte, Dephaforum, Seagen. F.D.B. is a principal investigator for Novartis, F.Hoffmann‐LaRoche Ltd, BMS, Ignyta Operating INC., Merck Sharp & Dohme Spa, Kymab, Pfizer, Tesaro, MSD, MedImmune LCC, Exelixis Inc., LOXO Oncology Incorporated, DAICHI SANKIO Dev. Limited, Basilea Pharmaceutica International AG, Janssen‐Cilag International NV, Merck KGAA. G.P. is a recipient of travel expenses / honoraria from Lilly, AstraZeneca, Exact Sciences, Gilead. G.P is part of the advisory board of ADSBiotec. No potential conflicts of interest were disclosed by the other authors.

## Supporting information


**Data S1:** Supporting Information

## Data Availability

All the datasets considered in the study are stored in public repositories. GSE132305, GSE32225, GSE26566, GSE89749, GSE32879 and GSE57555 were obtained from Gene Expression Omnibus (GEO). TCGA‐CHOL was obtained from Firebrowse. EGAD00001001693 was obtained from EGA repository after the accession permission released by ICGC consortium (application number: DACO‐6992).
