# Supplementary material for: Comprehensive transcriptomic analysis to identify biological and clinical differences in cholangiocarcinoma
Source: Cancer Med. 2023 Mar 20;12(8):10156–68. doi: 10.1002/cam4.5719 (PMC10166943; doi:10.1002/cam4.5719)
Supplement: Supplementary file 1 — Data S1: Supporting Information [file CAM4-12-10156-s001.pdf]

Comprehensive transcriptomic analysis to identify biological and clinical differences in cholangiocarcinoma.

Marco Silvestri[1,2], Trung Nghia Vu[3], Federico Nichetti[4,5], Monica Niger[4], Serena Di Cosimo[1], Filippo De Braud[4], Giancarlo Pruneri[6], Yudi Pawitan[3], Stefano Calza[2] ^*, Vera Cappelletti[1] ^*.

[1] Department of Applied Research and Technological Development, Fondazione IRCCS Istituto Nazionale dei Tumori di Milano, Via Giacomo Venezian 1, 20133 Milan, Italy.

[2] Unit of Biostatistics, Department of Molecular and Translational Medicine, University of Brescia, 25125 Brescia, Italy.

[3] Department of Medical Epidemiology and Biostatistics, Karolinska Institutet, Nobels väg 12A, 171 65 Solna (Stockholm), Sweden.

[4] Department of Medical Oncology, Fondazione IRCCS Istituto Nazionale dei Tumori di Milano, Via Giacomo Venezian 1, 20133 Milan, Italy

[5] Computational Oncology Group, Molecular Precision Oncology Program, National Center for Tumor Diseases (NCT) and German Cancer Research Center (DKFZ), Im Neuenheimer Feld 280, 69120 Heidelberg, Germany

[6] Department Pathology and Laboratory Medicine, Fondazione IRCCS Istituto Nazionale dei Tumori, Via Giacomo Venezian 1, 20133 Milan, Italy.

^ Co-last author

*** Co-Corresponding author**

Stefano Calza; stefano.calza@unibs.it ; +39 030 3717713

Vera Cappelletti; vera.cappelletti@istitutotumori.mi.it; +39 02 23902700

Table of contents

Supplementary tables (pages 2-12)

Supplementary figures (pages 13-24)

Supplementary Tables

Supplementary table 1-2. *Samples distribution within subgroups of ICC and ECC cohorts.*

ICC cohort				ECC cohort		
**batch**	**Type**	**Cluster**		**batch**	**Type**	**Cluster**
GSE26566	ICC	A		GSE89749	ECC	A
GSE26566	ICC	A		GSE89749	ECC	A
GSE26566	ICC	A		GSE89749	ECC	A
GSE26566	ICC	A		GSE89749	ECC	A
GSE26566	ICC	A		GSE89749	ECC	A
GSE26566	ICC	A		GSE89749	ECC	A
GSE26566	ICC	A		GSE89749	ECC	A
GSE26566	ICC	A		GSE89749	ECC	A
GSE26566	ICC	A		GSE89749	ECC	A
GSE26566	ICC	A		GSE132305	ECC	A
GSE26566	ICC	A		GSE132305	ECC	A
GSE26566	ICC	A		GSE132305	ECC	A
GSE26566	ICC	A		GSE132305	ECC	A
GSE26566	ICC	A		GSE132305	ECC	A
GSE26566	ICC	A		GSE132305	ECC	A
GSE26566	ICC	A		GSE132305	ECC	A
GSE26566	ICC	A		GSE132305	ECC	A
GSE26566	ICC	A		GSE132305	ECC	A
GSE26566	ICC	A		GSE132305	ECC	A
GSE26566	ICC	A		GSE132305	ECC	A
GSE26566	ICC	A		GSE132305	ECC	A
GSE26566	ICC	A		GSE132305	ECC	A
GSE26566	ICC	A		GSE132305	ECC	A
GSE26566	ICC	A		GSE132305	ECC	A
GSE26566	ICC	A		GSE132305	ECC	A
GSE26566	ICC	A		GSE132305	ECC	A
GSE26566	ICC	A		GSE132305	ECC	A
GSE26566	ICC	A		GSE132305	ECC	A
GSE26566	ICC	A		GSE132305	ECC	A
GSE26566	ICC	A		GSE132305	ECC	A
GSE26566	ICC	A		GSE132305	ECC	A
GSE26566	ICC	A		GSE132305	ECC	A
GSE26566	ICC	A		GSE132305	ECC	A
GSE26566	ICC	A		GSE132305	ECC	A
GSE26566	ICC	A		GSE132305	ECC	A
GSE26566	ICC	A		GSE132305	ECC	A

**GSE26566**	**ICC**	**A**		**GSE132305**	**ECC**	**A**
**GSE26566**	**ICC**	**A**		**GSE132305**	**ECC**	**A**
**GSE26566**	**ICC**	**A**		**GSE132305**	**ECC**	**A**
**GSE26566**	**ICC**	**A**		**GSE132305**	**ECC**	**A**
**GSE26566**	**ICC**	**A**		**GSE132305**	**ECC**	**A**
**GSE26566**	**ICC**	**A**		**GSE132305**	**ECC**	**A**
**GSE26566**	**ICC**	**A**		**GSE132305**	**ECC**	**A**
**GSE26566**	**ICC**	**A**		**GSE132305**	**ECC**	**A**
**GSE26566**	**ICC**	**A**		**GSE132305**	**ECC**	**A**
**GSE26566**	**ICC**	**A**		**GSE132305**	**ECC**	**A**
**GSE26566**	**ICC**	**A**		**GSE132305**	**ECC**	**A**
**GSE32225**	**ICC**	**A**		**GSE132305**	**ECC**	**A**
**GSE32225**	**ICC**	**A**		**GSE132305**	**ECC**	**A**
**GSE32225**	**ICC**	**A**		**GSE132305**	**ECC**	**A**
**GSE32225**	**ICC**	**A**		**GSE132305**	**ECC**	**A**
**GSE32225**	**ICC**	**A**		**GSE132305**	**ECC**	**A**
**GSE32225**	**ICC**	**A**		**GSE132305**	**ECC**	**A**
**GSE32225**	**ICC**	**A**		**GSE132305**	**ECC**	**A**
**GSE32225**	**ICC**	**A**		**GSE132305**	**ECC**	**A**
**GSE32225**	**ICC**	**A**		**GSE132305**	**ECC**	**A**
**GSE32225**	**ICC**	**A**		**GSE132305**	**ECC**	**A**
**GSE32225**	**ICC**	**A**		**GSE132305**	**ECC**	**A**
**GSE32225**	**ICC**	**A**		**GSE132305**	**ECC**	**A**
**GSE32225**	**ICC**	**A**		**GSE132305**	**ECC**	**A**
**GSE32225**	**ICC**	**A**		**GSE132305**	**ECC**	**A**
**GSE32225**	**ICC**	**A**		**GSE132305**	**ECC**	**A**
**GSE32225**	**ICC**	**A**		**GSE132305**	**ECC**	**A**
**GSE32225**	**ICC**	**A**		**GSE132305**	**ECC**	**A**
**GSE32225**	**ICC**	**A**		**GSE132305**	**ECC**	**A**
**GSE32225**	**ICC**	**A**		**GSE132305**	**ECC**	**A**
**GSE32225**	**ICC**	**A**		**GSE132305**	**ECC**	**A**
**GSE32225**	**ICC**	**A**		**GSE132305**	**ECC**	**A**
**GSE32225**	**ICC**	**A**		**GSE132305**	**ECC**	**A**
**GSE32225**	**ICC**	**A**		**GSE132305**	**ECC**	**A**
**GSE32225**	**ICC**	**A**		**GSE132305**	**ECC**	**A**
**GSE32225**	**ICC**	**A**		**GSE132305**	**ECC**	**A**
**GSE32225**	**ICC**	**A**		**GSE132305**	**ECC**	**A**
**GSE32225**	**ICC**	**A**		**GSE132305**	**ECC**	**A**
**GSE32225**	**ICC**	**A**		**GSE132305**	**ECC**	**A**
**GSE32879**	**ICC**	**A**		**GSE132305**	**ECC**	**A**
**GSE32879**	**ICC**	**A**		**GSE132305**	**ECC**	**A**
**GSE32879**	**ICC**	**A**		**GSE132305**	**ECC**	**A**
**GSE57555**	**ICC**	**A**		**GSE132305**	**ECC**	**A**

**GSE57555**	**ICC**	**A**		**GSE132305**	**ECC**	**A**
**GSE89749**	**ICC**	**A**		**GSE132305**	**ECC**	**A**
**GSE89749**	**ICC**	**A**		**GSE132305**	**ECC**	**A**
**GSE89749**	**ICC**	**A**		**GSE132305**	**ECC**	**A**
**GSE89749**	**ICC**	**A**		**GSE132305**	**ECC**	**A**
**GSE89749**	**ICC**	**A**		**GSE132305**	**ECC**	**A**
**GSE89749**	**ICC**	**A**		**GSE132305**	**ECC**	**A**
**GSE89749**	**ICC**	**A**		**GSE132305**	**ECC**	**A**
**GSE89749**	**ICC**	**A**		**TCGA_CHOL**	**ECC**	**A**
**GSE89749**	**ICC**	**A**		**TCGA_CHOL**	**ECC**	**A**
**GSE89749**	**ICC**	**A**		**GSE89749**	**ECC**	**B**
**TCGA_CHOL**	**ICC**	**A**		**GSE89749**	**ECC**	**B**
**TCGA_CHOL**	**ICC**	**A**		**GSE132305**	**ECC**	**B**
**TCGA_CHOL**	**ICC**	**A**		**GSE132305**	**ECC**	**B**
**TCGA_CHOL**	**ICC**	**A**		**GSE132305**	**ECC**	**B**
**TCGA_CHOL**	**ICC**	**A**		**GSE132305**	**ECC**	**B**
**TCGA_CHOL**	**ICC**	**A**		**GSE132305**	**ECC**	**B**
**GSE26566**	**ICC**	**B**		**GSE132305**	**ECC**	**B**
**GSE26566**	**ICC**	**B**		**GSE132305**	**ECC**	**B**
**GSE26566**	**ICC**	**B**		**GSE132305**	**ECC**	**B**
**GSE26566**	**ICC**	**B**		**GSE132305**	**ECC**	**B**
**GSE26566**	**ICC**	**B**		**GSE132305**	**ECC**	**B**
**GSE26566**	**ICC**	**B**		**GSE132305**	**ECC**	**B**
**GSE26566**	**ICC**	**B**		**GSE132305**	**ECC**	**B**
**GSE26566**	**ICC**	**B**		**GSE132305**	**ECC**	**B**
**GSE26566**	**ICC**	**B**		**GSE132305**	**ECC**	**B**
**GSE26566**	**ICC**	**B**		**GSE132305**	**ECC**	**B**
**GSE26566**	**ICC**	**B**		**GSE132305**	**ECC**	**B**
**GSE26566**	**ICC**	**B**		**GSE132305**	**ECC**	**B**
**GSE26566**	**ICC**	**B**		**GSE132305**	**ECC**	**B**
**GSE26566**	**ICC**	**B**		**GSE132305**	**ECC**	**B**
**GSE26566**	**ICC**	**B**		**GSE132305**	**ECC**	**B**
**GSE26566**	**ICC**	**B**		**GSE132305**	**ECC**	**B**
**GSE26566**	**ICC**	**B**		**GSE132305**	**ECC**	**B**
**GSE26566**	**ICC**	**B**		**GSE132305**	**ECC**	**B**
**GSE26566**	**ICC**	**B**		**GSE132305**	**ECC**	**B**
**GSE26566**	**ICC**	**B**		**GSE132305**	**ECC**	**B**
**GSE26566**	**ICC**	**B**		**GSE132305**	**ECC**	**B**
**GSE26566**	**ICC**	**B**		**GSE132305**	**ECC**	**B**
**GSE26566**	**ICC**	**B**		**GSE132305**	**ECC**	**B**
**GSE26566**	**ICC**	**B**		**GSE132305**	**ECC**	**B**
**GSE32225**	**ICC**	**B**		**GSE132305**	**ECC**	**B**
**GSE32225**	**ICC**	**B**		**GSE132305**	**ECC**	**B**

**GSE32225**	**ICC**	**B**		**TCGA_CHOL**	**ECC**	**B**
**GSE32225**	**ICC**	**B**		**GSE89749**	**ECC**	**C**
**GSE32225**	**ICC**	**B**		**GSE132305**	**ECC**	**C**
**GSE32225**	**ICC**	**B**		**GSE132305**	**ECC**	**C**
**GSE32225**	**ICC**	**B**		**GSE132305**	**ECC**	**C**
**GSE32225**	**ICC**	**B**		**GSE132305**	**ECC**	**C**
**GSE32225**	**ICC**	**B**		**GSE132305**	**ECC**	**C**
**GSE32225**	**ICC**	**B**		**GSE132305**	**ECC**	**C**
**GSE32879**	**ICC**	**B**		**GSE132305**	**ECC**	**C**
**GSE32879**	**ICC**	**B**		**GSE132305**	**ECC**	**C**
**GSE32879**	**ICC**	**B**		**GSE132305**	**ECC**	**C**
**GSE57555**	**ICC**	**B**		**GSE132305**	**ECC**	**C**
**GSE57555**	**ICC**	**B**		**GSE132305**	**ECC**	**C**
**GSE89749**	**ICC**	**B**		**GSE132305**	**ECC**	**C**
**GSE89749**	**ICC**	**B**		**GSE132305**	**ECC**	**C**
**GSE89749**	**ICC**	**B**		**GSE132305**	**ECC**	**C**
**GSE89749**	**ICC**	**B**		**GSE132305**	**ECC**	**C**
**GSE89749**	**ICC**	**B**		**GSE132305**	**ECC**	**C**
**GSE89749**	**ICC**	**B**		**GSE132305**	**ECC**	**C**
**TCGA_CHOL**	**ICC**	**B**		**GSE132305**	**ECC**	**C**
**TCGA_CHOL**	**ICC**	**B**		**GSE132305**	**ECC**	**C**
**TCGA_CHOL**	**ICC**	**B**		**GSE132305**	**ECC**	**C**
**TCGA_CHOL**	**ICC**	**B**		**GSE132305**	**ECC**	**C**
**TCGA_CHOL**	**ICC**	**B**		**GSE132305**	**ECC**	**C**
**GSE26566**	**ICC**	**C**		**GSE132305**	**ECC**	**C**
**GSE26566**	**ICC**	**C**		**GSE132305**	**ECC**	**C**
**GSE26566**	**ICC**	**C**		**GSE132305**	**ECC**	**C**
**GSE26566**	**ICC**	**C**		**GSE132305**	**ECC**	**C**
**GSE26566**	**ICC**	**C**		**GSE132305**	**ECC**	**C**
**GSE26566**	**ICC**	**C**		**GSE132305**	**ECC**	**C**
**GSE26566**	**ICC**	**C**		**GSE132305**	**ECC**	**C**
**GSE26566**	**ICC**	**C**		**GSE132305**	**ECC**	**C**
**GSE26566**	**ICC**	**C**		**GSE132305**	**ECC**	**C**
**GSE26566**	**ICC**	**C**		**GSE132305**	**ECC**	**C**
**GSE26566**	**ICC**	**C**		**GSE132305**	**ECC**	**C**
**GSE26566**	**ICC**	**C**		**GSE132305**	**ECC**	**C**
**GSE26566**	**ICC**	**C**		**GSE132305**	**ECC**	**C**
**GSE26566**	**ICC**	**C**		**GSE132305**	**ECC**	**C**
**GSE26566**	**ICC**	**C**		**GSE132305**	**ECC**	**C**
**GSE26566**	**ICC**	**C**		**GSE132305**	**ECC**	**C**
**GSE26566**	**ICC**	**C**		**GSE132305**	**ECC**	**C**
**GSE26566**	**ICC**	**C**		**GSE132305**	**ECC**	**C**
**GSE26566**	**ICC**	**C**		**GSE132305**	**ECC**	**C**

**GSE26566**	**ICC**	**C**		**GSE132305**	**ECC**	**C**
**GSE26566**	**ICC**	**C**		**GSE132305**	**ECC**	**C**
**GSE26566**	**ICC**	**C**		**GSE132305**	**ECC**	**C**
**GSE26566**	**ICC**	**C**		**GSE132305**	**ECC**	**C**
**GSE26566**	**ICC**	**C**		**GSE132305**	**ECC**	**C**
**GSE26566**	**ICC**	**C**		**GSE132305**	**ECC**	**C**
**GSE26566**	**ICC**	**C**		**GSE132305**	**ECC**	**C**
**GSE26566**	**ICC**	**C**		**GSE132305**	**ECC**	**C**
**GSE26566**	**ICC**	**C**		**GSE132305**	**ECC**	**C**
**GSE26566**	**ICC**	**C**		**GSE132305**	**ECC**	**C**
**GSE26566**	**ICC**	**C**		**GSE132305**	**ECC**	**C**
**GSE26566**	**ICC**	**C**		**GSE132305**	**ECC**	**C**
**GSE26566**	**ICC**	**C**		**GSE132305**	**ECC**	**C**
**GSE32225**	**ICC**	**C**		**GSE132305**	**ECC**	**C**
**GSE32225**	**ICC**	**C**		**GSE132305**	**ECC**	**C**
**GSE32225**	**ICC**	**C**		**GSE132305**	**ECC**	**C**
**GSE32225**	**ICC**	**C**		**GSE132305**	**ECC**	**C**
**GSE32225**	**ICC**	**C**		**GSE132305**	**ECC**	**C**
**GSE32225**	**ICC**	**C**		**GSE132305**	**ECC**	**C**
**GSE32225**	**ICC**	**C**		**TCGA_CHOL**	**ECC**	**C**
**GSE32225**	**ICC**	**C**		**GSE89749**	**ECC**	**D**
**GSE32225**	**ICC**	**C**		**GSE89749**	**ECC**	**D**
**GSE32225**	**ICC**	**C**		**GSE89749**	**ECC**	**D**
**GSE32225**	**ICC**	**C**		**GSE132305**	**ECC**	**D**
**GSE32225**	**ICC**	**C**		**GSE132305**	**ECC**	**D**
**GSE32225**	**ICC**	**C**		**GSE132305**	**ECC**	**D**
**GSE32225**	**ICC**	**C**		**GSE132305**	**ECC**	**D**
**GSE32225**	**ICC**	**C**		**GSE132305**	**ECC**	**D**
**GSE32225**	**ICC**	**C**		**GSE132305**	**ECC**	**D**
**GSE32225**	**ICC**	**C**		**GSE132305**	**ECC**	**D**
**GSE32225**	**ICC**	**C**		**GSE132305**	**ECC**	**D**
**GSE32225**	**ICC**	**C**		**GSE132305**	**ECC**	**D**
**GSE32225**	**ICC**	**C**		**GSE132305**	**ECC**	**D**
**GSE32225**	**ICC**	**C**		**GSE132305**	**ECC**	**D**
**GSE32225**	**ICC**	**C**		**GSE132305**	**ECC**	**D**
**GSE32225**	**ICC**	**C**		**GSE132305**	**ECC**	**D**
**GSE32225**	**ICC**	**C**		**TCGA_CHOL**	**ECC**	**D**
**GSE32225**	**ICC**	**C**		**TCGA_CHOL**	**ECC**	**D**
**GSE32225**	**ICC**	**C**				
**GSE32225**	**ICC**	**C**				
**GSE32225**	**ICC**	**C**				
**GSE32225**	**ICC**	**C**				
**GSE32225**	**ICC**	**C**				

GSE32225	ICC	C				
GSE32225	ICC	C				
GSE32225	ICC	C				
GSE32225	ICC	C				
GSE32225	ICC	C				
GSE32225	ICC	C				
GSE32225	ICC	C				
GSE32225	ICC	C				
GSE32225	ICC	C				
GSE32225	ICC	C				
GSE32225	ICC	C				
GSE32225	ICC	C				
GSE32225	ICC	C				
GSE32225	ICC	C				
GSE32225	ICC	C				
GSE32225	ICC	C				
GSE32225	ICC	C				
GSE32225	ICC	C				
GSE32225	ICC	C				
GSE32225	ICC	C				
GSE32225	ICC	C				
GSE32225	ICC	C				
GSE32225	ICC	C				
GSE32225	ICC	C				
GSE32225	ICC	C				
GSE32225	ICC	C				
GSE32225	ICC	C				
GSE32225	ICC	C				
GSE32225	ICC	C				
GSE32225	ICC	C				
GSE32225	ICC	C				
GSE32225	ICC	C				
GSE32225	ICC	C				
GSE32225	ICC	C				
GSE32225	ICC	C				
GSE32225	ICC	C				
GSE32225	ICC	C				
GSE32225	ICC	C				
GSE32225	ICC	C				
GSE32879	ICC	C				
GSE32879	ICC	C				
GSE32879	ICC	C				
GSE32879	ICC	C				

**GSE32879**	**ICC**	**C**				
**GSE32879**	**ICC**	**C**				
**GSE32879**	**ICC**	**C**				
**GSE32879**	**ICC**	**C**				
**GSE32879**	**ICC**	**C**				
**GSE32879**	**ICC**	**C**				
**GSE57555**	**ICC**	**C**				
**GSE57555**	**ICC**	**C**				
**GSE89749**	**ICC**	**C**				
**GSE89749**	**ICC**	**C**				
**GSE89749**	**ICC**	**C**				
**GSE89749**	**ICC**	**C**				
**GSE89749**	**ICC**	**C**				
**GSE89749**	**ICC**	**C**				
**GSE89749**	**ICC**	**C**				
**GSE89749**	**ICC**	**C**				
**GSE89749**	**ICC**	**C**				
**GSE89749**	**ICC**	**C**				
**GSE89749**	**ICC**	**C**				
**GSE89749**	**ICC**	**C**				
**GSE89749**	**ICC**	**C**				
**GSE89749**	**ICC**	**C**				
**GSE89749**	**ICC**	**C**				
**GSE89749**	**ICC**	**C**				
**GSE89749**	**ICC**	**C**				
**GSE89749**	**ICC**	**C**				
**GSE89749**	**ICC**	**C**				
**GSE89749**	**ICC**	**C**				
**GSE89749**	**ICC**	**C**				
**GSE89749**	**ICC**	**C**				
**GSE89749**	**ICC**	**C**				
**GSE89749**	**ICC**	**C**				
**GSE89749**	**ICC**	**C**				
**GSE89749**	**ICC**	**C**				
**GSE89749**	**ICC**	**C**				
**TCGA_CHOL**	**ICC**	**C**				
**TCGA_CHOL**	**ICC**	**C**				
**TCGA_CHOL**	**ICC**	**C**				
**TCGA_CHOL**	**ICC**	**C**				
**TCGA_CHOL**	**ICC**	**C**				
**TCGA_CHOL**	**ICC**	**C**				
**TCGA_CHOL**	**ICC**	**C**				
**TCGA_CHOL**	**ICC**	**C**				

**TCGA_CHOL**	**ICC**	**C**				
**TCGA_CHOL**	**ICC**	**C**				
**TCGA_CHOL**	**ICC**	**C**				
**TCGA_CHOL**	**ICC**	**C**				
**TCGA_CHOL**	**ICC**	**C**				
**TCGA_CHOL**	**ICC**	**C**				
**TCGA_CHOL**	**ICC**	**C**				
**TCGA_CHOL**	**ICC**	**C**				
**TCGA_CHOL**	**ICC**	**C**				
**TCGA_CHOL**	**ICC**	**C**				
**TCGA_CHOL**	**ICC**	**C**				
**GSE32225**	**ICC**	**D**				
**GSE32225**	**ICC**	**D**				
**GSE32225**	**ICC**	**D**				
**GSE32225**	**ICC**	**D**				
**GSE32225**	**ICC**	**D**				
**GSE32225**	**ICC**	**D**				
**GSE32225**	**ICC**	**D**				
**GSE32225**	**ICC**	**D**				
**GSE32225**	**ICC**	**D**				
**GSE32225**	**ICC**	**D**				
**GSE32225**	**ICC**	**D**				
**GSE32225**	**ICC**	**D**				
**GSE32225**	**ICC**	**D**				
**GSE32225**	**ICC**	**D**				
**GSE32225**	**ICC**	**D**				
**GSE32225**	**ICC**	**D**				
**GSE32225**	**ICC**	**D**				
**GSE32225**	**ICC**	**D**				
**GSE32225**	**ICC**	**D**				
**GSE32225**	**ICC**	**D**				
**GSE32225**	**ICC**	**D**				
**GSE32225**	**ICC**	**D**				
**GSE32225**	**ICC**	**D**				
**GSE32225**	**ICC**	**D**				
**GSE32225**	**ICC**	**D**				
**GSE32225**	**ICC**	**D**				
**GSE32225**	**ICC**	**D**				
**GSE32225**	**ICC**	**D**				
**GSE32225**	**ICC**	**D**				
**GSE32225**	**ICC**	**D**				
**GSE32225**	**ICC**	**D**				
**GSE32225**	**ICC**	**D**				

**GSE32225**	**ICC**	**D**				
**GSE32225**	**ICC**	**D**				
**GSE57555**	**ICC**	**D**				

Supplementary table 3. *Performance of predictors in ICC dataset*

ICC cohort

Fast unified random forest (rFSRC)

```
                         Sample size: 340
           Frequency of class labels: 85, 85, 85, 85
                     Number of trees: 500
           Forest terminal node size: 1
       Average no. of terminal nodes: 42.636
No. of variables tried at each split: 5
              Total no. of variables: 19
       Resampling used to grow trees: swor
    Resample size used to grow trees: 215
                            Analysis: RF-C
                              Family: class
                      Splitting rule: gini *random*
       Number of random split points: 10
                   (OOB) Brier score: 0.03955124
        (OOB) Normalized Brier score: 0.21093992
                           (OOB) AUC: 0.98787197
   (OOB) Requested performance error: 0.08235294, 0.03529412, 0.11764706, 0.10588235, 0.07058824

Confusion matrix:

          predicted
  observed  A  B  C  D class.error
         A 82  1  2  0      0.0353
         B  4 75  6  0      0.1176
         C  5  2 76  2      0.1059
         D  2  3  1 79      0.0706

      (OOB) Misclassification rate: 0.08235294
```

Support vector machine (SVM)

```
Confusion Matrix and Statistics

          Reference
Prediction  A  B  C  D
         A 39  2 21  1
         B  0 13  8  1
         C  0  2 33  8
         D  1  0  2  5

Overall Statistics

               Accuracy : 0.6618
                 95% CI : (0.5757, 0.7406)
    No Information Rate : 0.4706
    P-Value [Acc > NIR] : 5.417e-06

                  Kappa : 0.5086

 Mcnemar's Test P-Value : 2.321e-05

Statistics by Class:

                     Class: A Class: B Class: C Class: D
Sensitivity            0.9750  0.76471   0.5156  0.33333
Specificity            0.7500  0.92437   0.8611  0.97521
Pos Pred Value         0.6190  0.59091   0.7674  0.62500
Neg Pred Value         0.9863  0.96491   0.6667  0.92188
Prevalence             0.2941  0.12500   0.4706  0.11029
Detection Rate         0.2868  0.09559   0.2426  0.03676
Detection Prevalence   0.4632  0.16176   0.3162  0.05882
Balanced Accuracy      0.8625  0.84454   0.6884  0.65427
```

K-nearest neighbors (KNN)

```
Confusion Matrix and Statistics

          Reference
Prediction  A  B  C  D
         A 39  2 21  1
         B  0 13  8  1
         C  0  2 33  8
         D  1  0  2  5

Overall Statistics

               Accuracy : 0.6618
                 95% CI : (0.5757, 0.7406)
    No Information Rate : 0.4706
    P-Value [Acc > NIR] : 5.417e-06

                  Kappa : 0.5086

 Mcnemar's Test P-Value : 2.321e-05

Statistics by Class:

                     Class: A Class: B Class: C Class: D
Sensitivity            0.9750  0.76471   0.5156  0.33333
Specificity            0.7500  0.92437   0.8611  0.97521
Pos Pred Value         0.6190  0.59091   0.7674  0.62500
Neg Pred Value         0.9863  0.96491   0.6667  0.92188
Prevalence             0.2941  0.12500   0.4706  0.11029
Detection Rate         0.2868  0.09559   0.2426  0.03676
Detection Prevalence   0.4632  0.16176   0.3162  0.05882
Balanced Accuracy      0.8625  0.84454   0.6884  0.65427
```

Supplementary table 4. *Performance of predictors in ECC dataset*

ECC cohort

Fast unified random forest (rFSRC)

```
                         Sample size: 203
           Frequency of class labels: 51, 51, 51, 50
                     Number of trees: 500
           Forest terminal node size: 1
       Average no. of terminal nodes: 25.94
No. of variables tried at each split: 5
              Total no. of variables: 21
       Resampling used to grow trees: swor
    Resample size used to grow trees: 128
                            Analysis: RF-C
                              Family: class
                      Splitting rule: gini *random*
       Number of random split points: 10
                  (OOB) Brier score: 0.03720509
       (OOB) Normalized Brier score: 0.19842717
                          (OOB) AUC: 0.99207612
   (OOB) Requested performance error: 0.07389163, 0.17647059, 0.01960784, 0.07843137, 0.02

Confusion matrix:

          predicted
  observed  A  B  C  D class.error
         A 42  4  4  1      0.1765
         B  0 50  1  0      0.0196
         C  3  1 47  0      0.0784
         D  0  0  1 49      0.0200

      (OOB) Misclassification rate: 0.07389163
```

Support vector machine (SVM)

```
Confusion Matrix and Statistics

          Reference
Prediction  A  B  C  D
         A 31  1 11  0
         B  1  7  4  0
         C  2  2  9  0
         D  2  0  3  7

Overall Statistics

               Accuracy : 0.675
                 95% CI : (0.5611, 0.7755)
    No Information Rate : 0.45
    P-Value [Acc > NIR] : 4.098e-05

                  Kappa : 0.5159

 Mcnemar's Test P-Value : NA

Statistics by Class:

                     Class: A Class: B Class: C Class: D
Sensitivity            0.8611   0.7000   0.3333   1.0000
Specificity            0.7273   0.9286   0.9245   0.9315
Pos Pred Value         0.7209   0.5833   0.6923   0.5833
Neg Pred Value         0.8649   0.9559   0.7313   1.0000
Prevalence             0.4500   0.1250   0.3375   0.0875
Detection Rate         0.3875   0.0875   0.1125   0.0875
Detection Prevalence   0.5375   0.1500   0.1625   0.1500
Balanced Accuracy      0.7942   0.8143   0.6289   0.9658
```

K-nearest neighbors (KNN)

```
Confusion Matrix and Statistics

          Reference
Prediction  A  B  C  D
         A 27  0  5  2
         B  1  8  0  0
         C  8  1 22  0
         D  0  1  0  5

Overall Statistics

               Accuracy : 0.775
                 95% CI : (0.6679, 0.8609)
    No Information Rate : 0.45
    P-Value [Acc > NIR] : 3.084e-09

                  Kappa : 0.6577

 Mcnemar's Test P-Value : NA

Statistics by Class:

                     Class: A Class: B Class: C Class: D
Sensitivity            0.7500   0.8000   0.8148   0.7143
Specificity            0.8409   0.9857   0.8302   0.9863
Pos Pred Value         0.7941   0.8889   0.7097   0.8333
Neg Pred Value         0.8043   0.9718   0.8980   0.9730
Prevalence             0.4500   0.1250   0.3375   0.0875
Detection Rate         0.3375   0.1000   0.2750   0.0625
Detection Prevalence   0.4250   0.1125   0.3875   0.0750
Balanced Accuracy      0.7955   0.8929   0.8225   0.8503
```

Supplementary figures

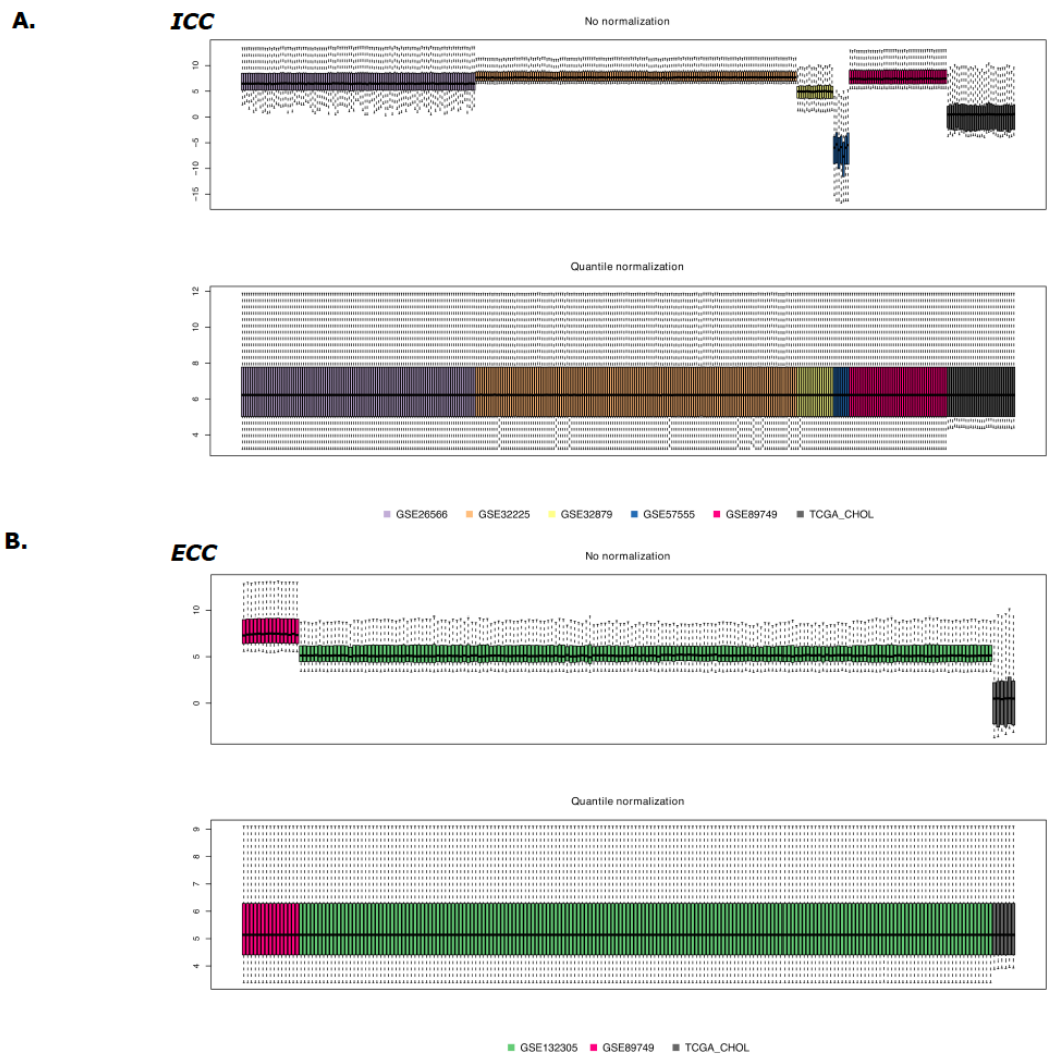

Figure S1. Integration and normalization of different dataset composing ICC and ECC training set. For both ICC (A) and ECC (B) samples, quantile normalization was performed to allow the correct integration of different dataset composing the training sets.

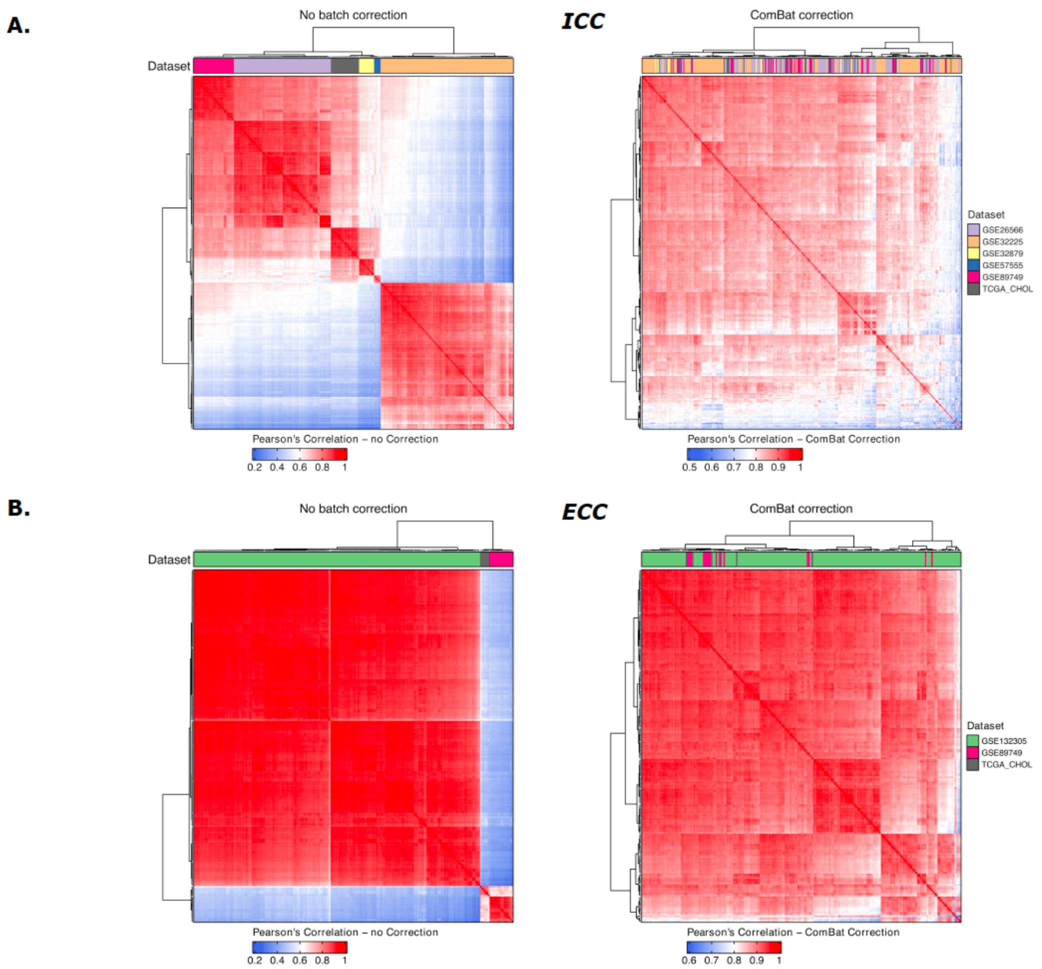

Figure S2. Batch effect correction by empirical byases model (ComBat). ICC (A) and ECC (B) training set were submitted to ComBat in order to remove the batch effect associated to different datasets. The heatmap reports the level of correlation (Prearson's) between samples.

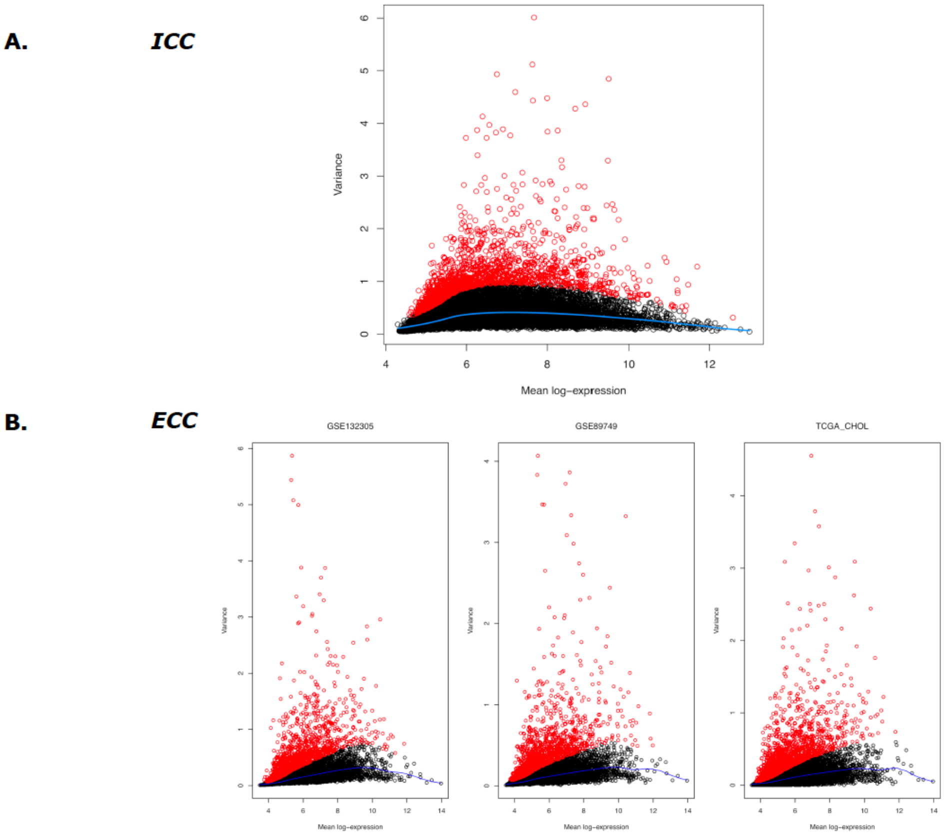

Figure S3. Gene filtering by mean expression and variance. For ICC (A) and ECC (B) training set, the highly variable genes based on their mean expression were selected. The plots report mean log-expression on the x-axis and variance level on the y-axis for each gene.

A. ICC cohort

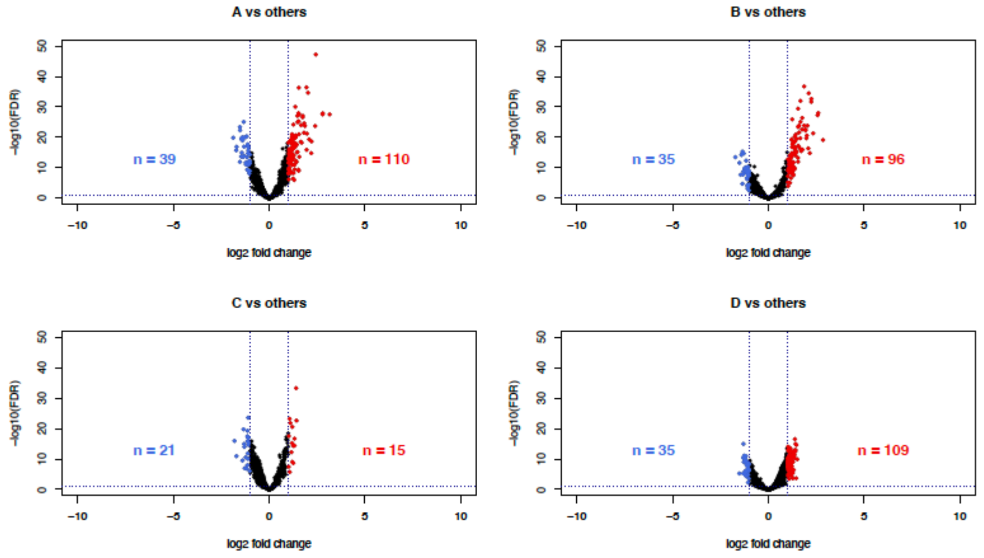

B. ECC cohort

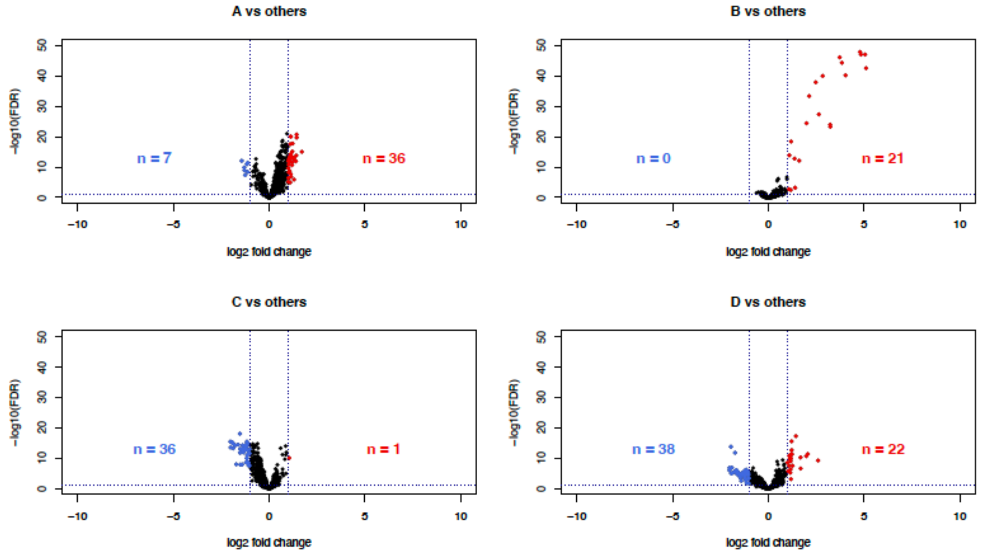

Figure S4. Class comparison by LIMMA. Differentially expressed genes between ICC (A) and ECC (B) subgroups were computed by LIMMA. The plot report log2 fold change on the x-axis and -log10(FDR) on the y-axis. Blue and red colors refer to downregulated and upregulated genes.

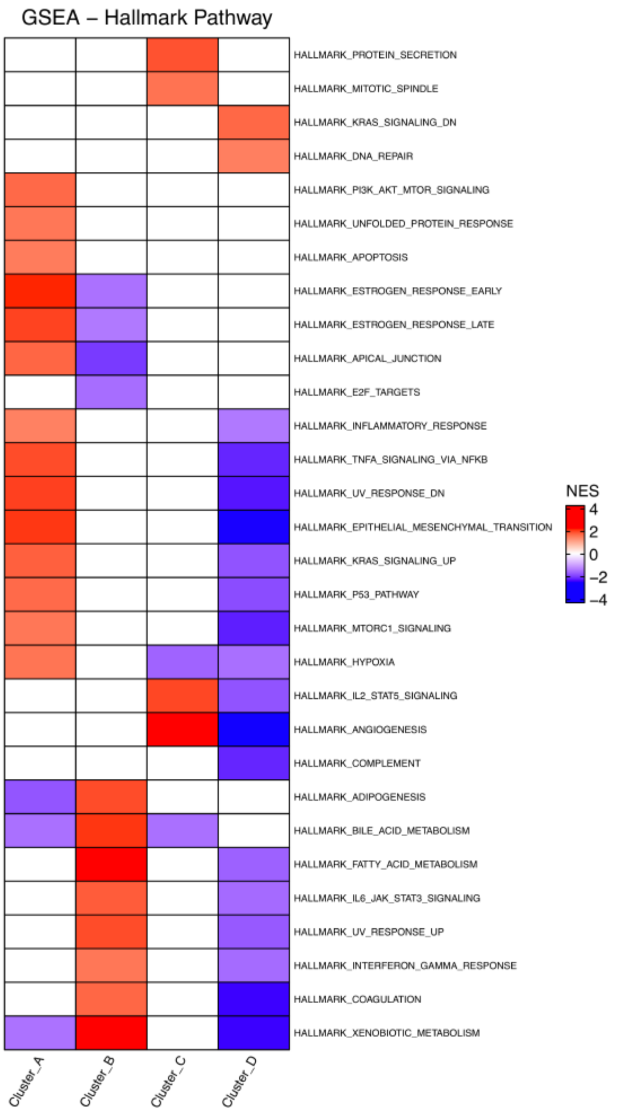

Figure S5. Gene set enrichment analysis (GSEA) on ICC cohort. The heatmap reports hallmark pathways on the rows and subgroups on the column. Blue and red colors refer to upregulated and downregulated pathways in term of nested enrichment score.

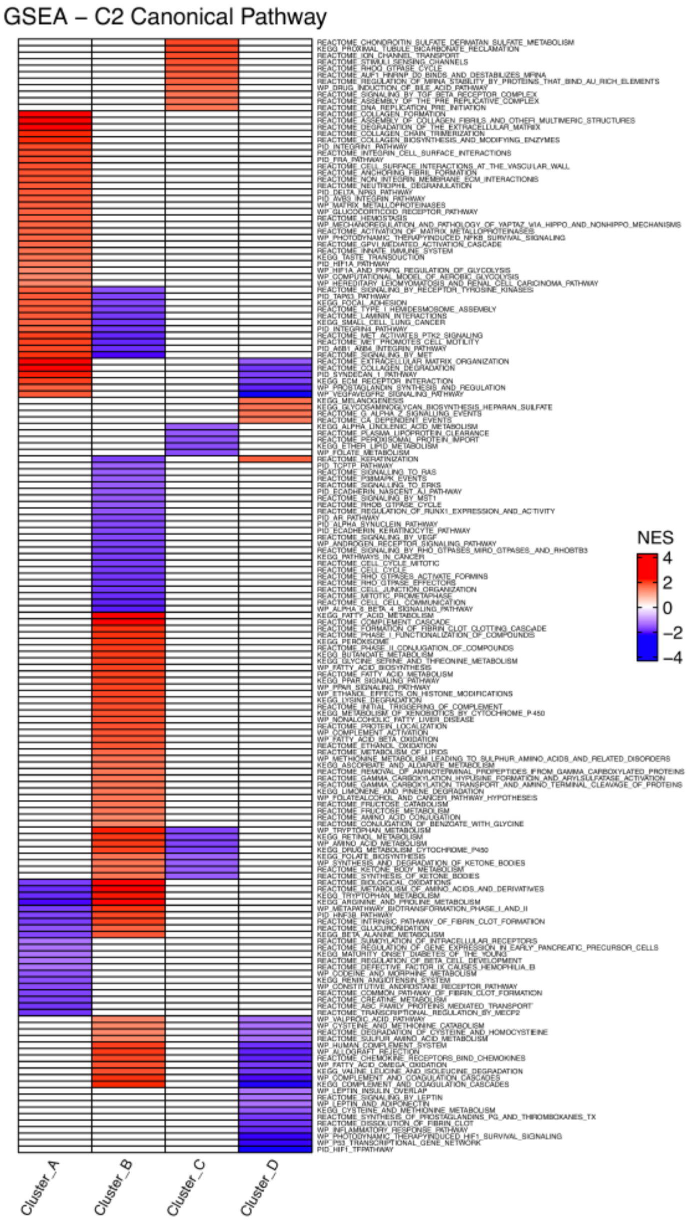

Figure S6. Gene set enrichment analysis (GSEA) on ICC cohort. The heatmap reports C2 canonical pathways on the rows and subgroups on the column. Blue and red colors refer to upregulated and downregulated pathways in term of nested enrichment score.

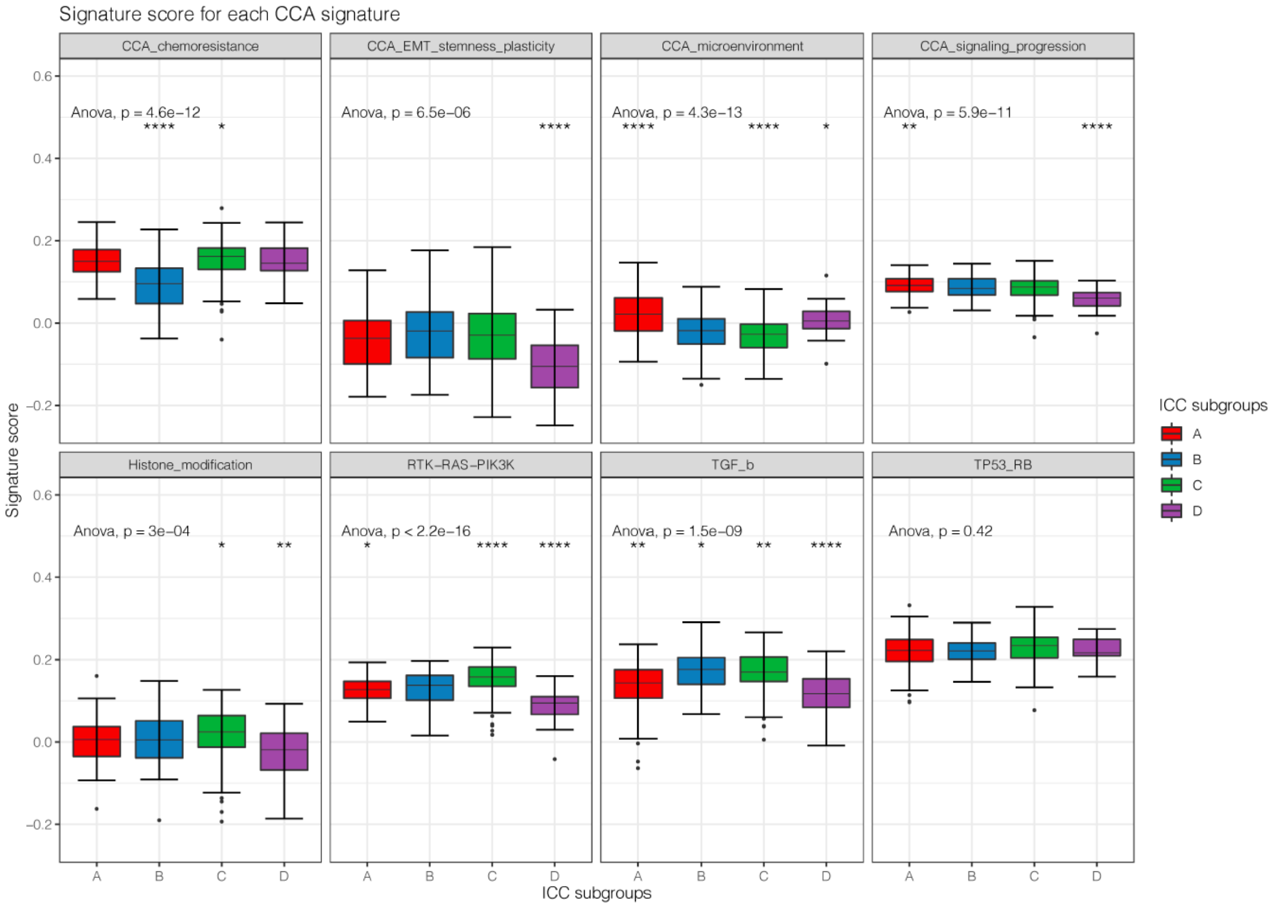

Figure S7. Gene signature scoring results in ICC cohort. The boxplots, coloured by subgroups, show signature scoring results obtained from Singscore for the selected cholangiocarcinoma signature.

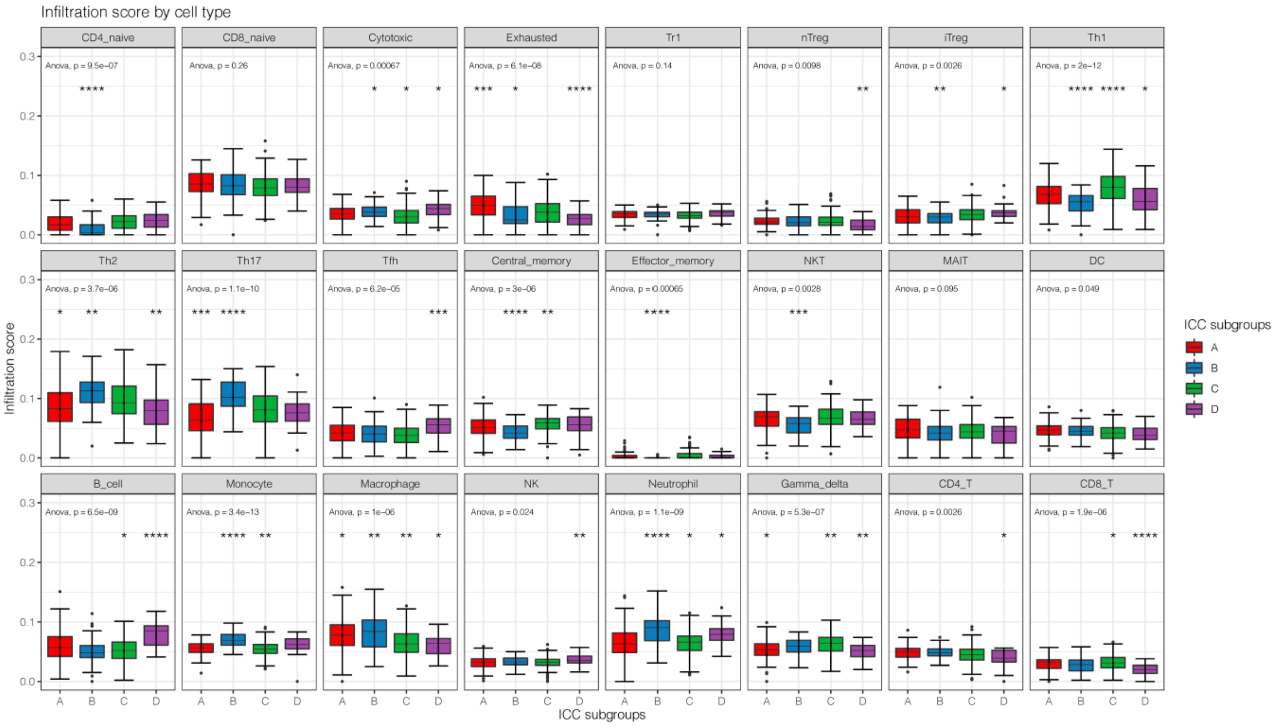

Figure S8. Analysis of immune cell infiltration in ICC cohort. The evaluation of immune cell components associated to each subgroup was performed using ImmuCellAI. The boxplots report the infiltration score of 24 immune cells for each subgroup.

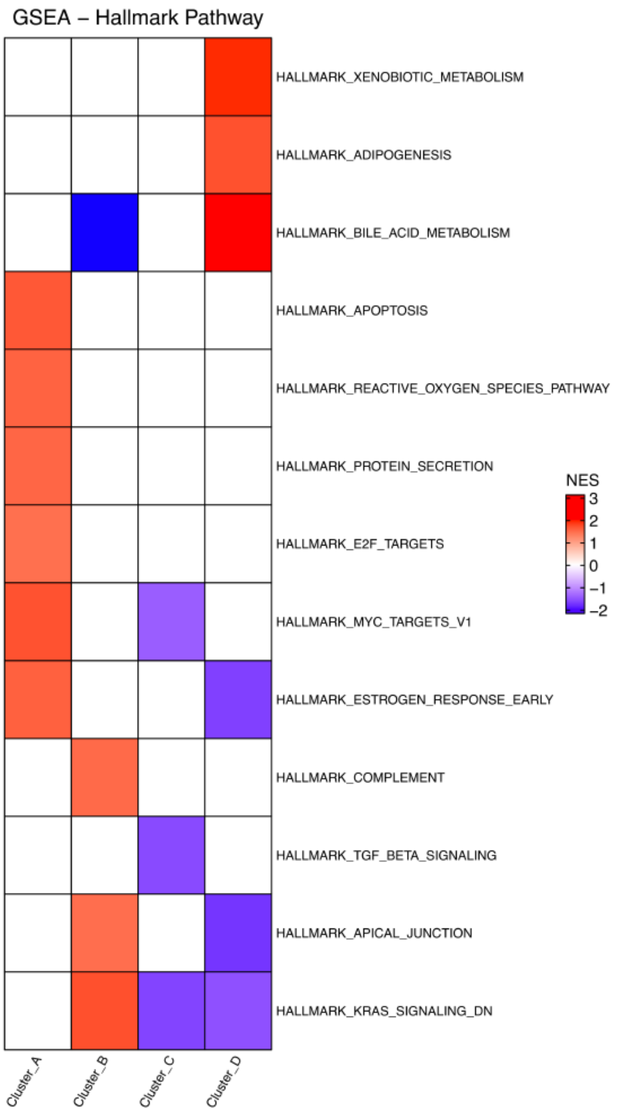

Figure S9. Gene set enrichment analysis (GSEA) on ECC cohort. The heatmap reports hallmark pathways on the rows and subgroups on the column. Blue and red colors refer to upregulated and downregulated pathways in term of nested enrichment score.

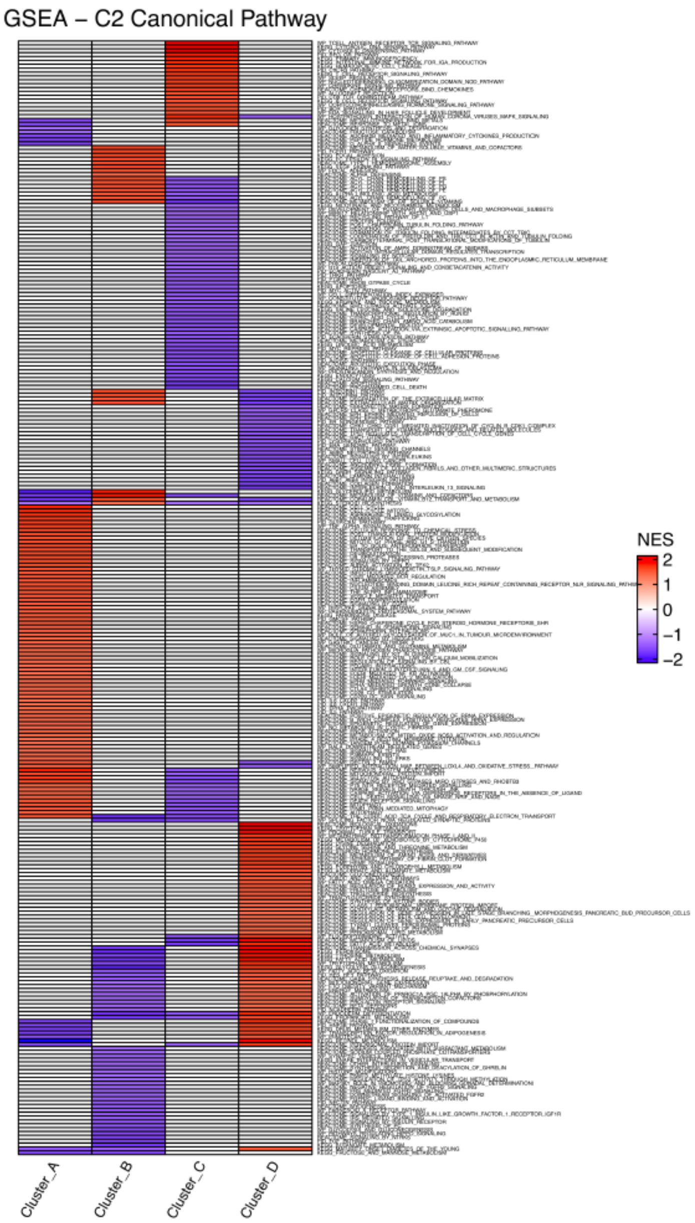

Figure S10. Gene set enrichment analysis (GSEA) on ECC cohort. The heatmap reports C2 canonical pathways on the rows and subgroups on the column. Blue and red colors refer to upregulated and downregulated pathways in term of nested enrichment score.

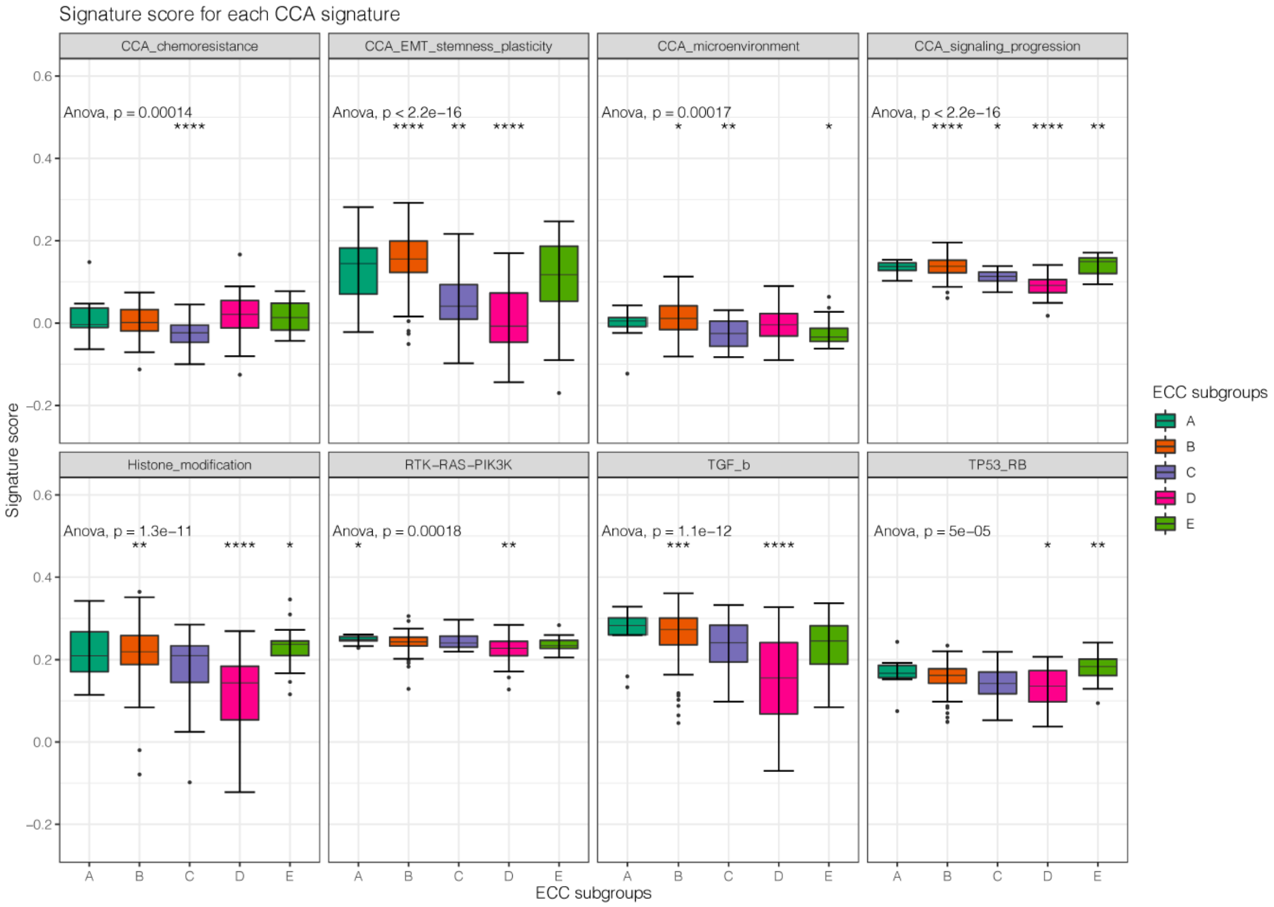

Figure S11. Gene signature scoring results in ECC cohort. The boxplots, coloured by subgroups, show signature scoring results obtained from Singscore for the selected cholangiocarcinoma signature.

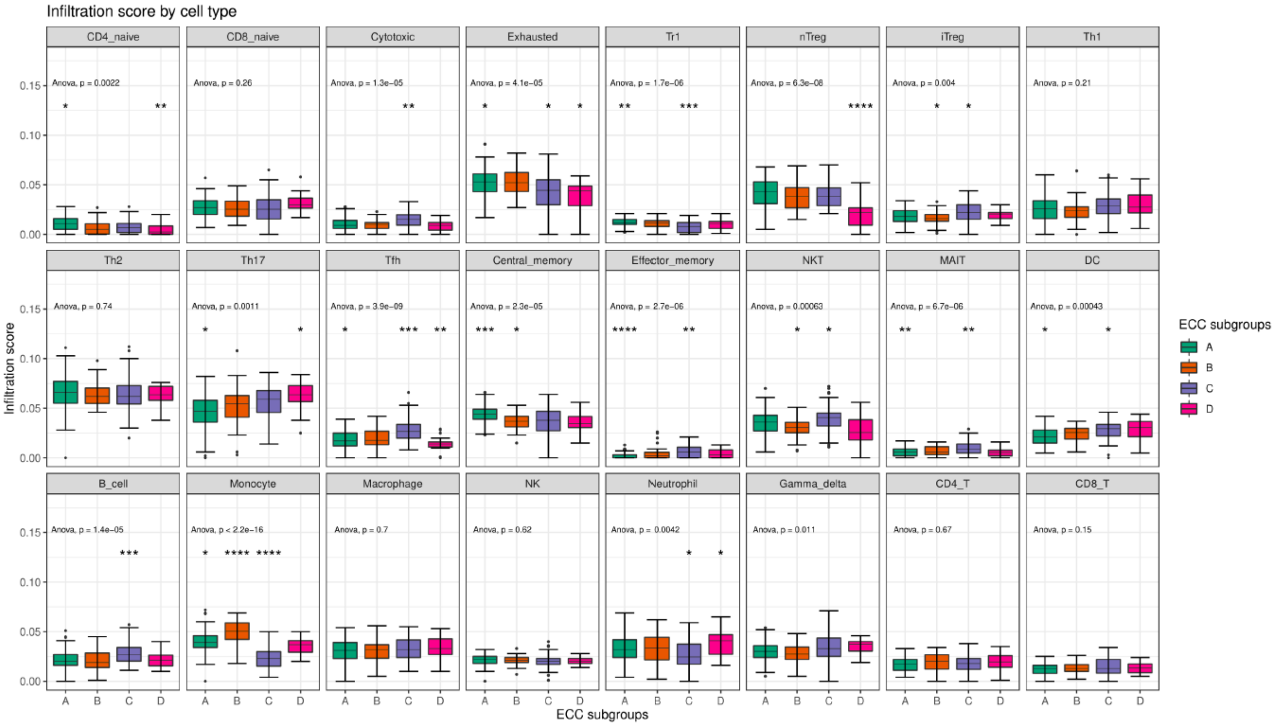

Figure S12. Analysis of immune cell infiltration in ECC cohort. The evaluation of immune cell components associated to each subgroup was performed using ImmuCellAI. The boxplots report the infiltration score of 24 immune cells for each subgroup.
